# Role of the uS9/yS16 C-terminal tail in translation initiation and elongation in *Saccharomyces cerevisiae*

**DOI:** 10.1093/nar/gky1180

**Published:** 2018-11-27

**Authors:** Supriya Jindal, Arnab Ghosh, Amra Ismail, Nishant Singh, Anton A Komar

**Affiliations:** Center for Gene Regulation in Health and Disease, Department of Biological, Geological and Environmental Sciences, Cleveland State University, Cleveland, OH 44115, USA

## Abstract

The small ribosomal subunit protein uS9 (formerly called rpS16 in *Saccharomyces cerevisiae*), has a long protruding C-terminal tail (CTT) that extends towards the mRNA cleft of the ribosome. The last C-terminal residue of uS9 is an invariably conserved, positively charged Arg that is believed to enhance interaction of the negatively charged initiator tRNA with the ribosome when the tRNA is base-paired to the AUG codon in the P-site. In order to more fully characterize the role of the uS9 CTT in eukaryotic translation, we tested how truncations, extensions and substitutions within the CTT affect initiation and elongation processes in *Saccharomyces cerevisiae*. We found that uS9 C-terminal residues are critical for efficient recruitment of the eIF2•GTP•Met-tRNA_i_^Met^ ternary complex to the ribosome and for its proper response to the presence of an AUG codon in the P-site during the scanning phase of initiation. These residues also regulate hydrolysis of the GTP in the eIF2•GTP•Met-tRNA_i_^Met^ complex to GDP and Pi. In addition, our data show that uS9 CTT modulates elongation fidelity. Therefore, we propose that uS9 CTT is critical for proper control of the complex interplay of events surrounding accommodation of initiator and elongator tRNAs in the P- and A-sites of the ribosome.

## INTRODUCTION

In both bacterial and eukaryotic systems, protein synthesis (translation) consists of four major steps: initiation, elongation, termination and ribosome recycling (for review, see (1–4)). The initiation step is the most complex phase in eukaryotes, and is the subject of fine-tuned and extensive control (1,4). Initiation leads to the placement of the initiator transfer RNA (tRNA) in the P-site of the ribosome, correctly base paired with the initiating AUG codon in the mRNA (1,4). The elongation phase involves sequential addition of the subsequent amino acids encoded by the mRNA to the nascent polypeptide chain. This phase is critical for maintaining the accuracy of protein synthesis as elongator aminoacyl (aa)-tRNAs must correctly ‘translate’ mRNA codons by bringing the correct amino acid to the ribosome. The ribosome has three major sites for tRNAs to bind: the peptidyl site (P-site), where the initiator tRNA binds and the peptidyl-tRNA is formed, the aminoacyl site (A-site), where all other (non-initiator) aminoacyl (aa)-tRNAs bind, and the (exit) E-site, where deacylated tRNAs leave the ribosome (2). Movement of the ribosome along the mRNA during translation involves displacement of tRNA-mRNA complexes from the A-site to the P-site and from the P-site to the E-site, all while ensuring maintenance of the correct reading frame (2,5). Translation termination is triggered by the entry of a stop codon into the ribosomal A-site. Ribosome recycling prepares the ribosome subunits for engagement in subsequent rounds of translation. While it is clear that integrity of the ribosomal A-, P- and E-sites and proper control of their occupancy is crucial for protein synthesis, the contribution of the environment and architecture of these sites is still not fully understood in molecular terms. The majority of mRNAs within eukaryotic cells are translated via the cap-dependent pathway, which begins with formation of a ternary complex (TC) comprised of the GTP-bound form of eukaryotic initiation factor 2 (eIF2) and initiator Met-tRNA_i_^Met^ (eIF2•GTP•Met-tRNA_i_^Met^) (1,4). The TC assembles with the 40S subunit and eIFs1, 1A, 3 and 5 to form the 43S pre-initiation complex (PIC). The 43S PIC binds to the 5′ capped end of the mRNA and scans the 5′-untranslated region (UTR) in search of an initiation codon. eIF1 blocks recognition of near cognate and cognate triplets in suboptimal context at the P-site. In cooperation with eIF1A, it also maintains a scanning competent open confirmation of the 43S PIC in which Met-tRNA_i_^Met^ is not fully engaged with the P-site (P_OUT_ state) (4). Recognition of the start codon leads to displacement of eIF1, followed by deep insertion of the anticodon stem loop (ASL) of Met-tRNA_i_^Met^ into the P-site (P_IN_ state). This results in a closed conformation (characteristic for the 48S complex) and eIF5-stimulated GTP hydrolysis and Pi release (4). Subsequently, eIF5B-mediated subunit joining leads to formation of the elongation competent 80S ribosome (3,4). The P-site of the 80S complex hosts the Met-tRNA_i_^Met^ with its anticodon base paired to the start codon in the mRNA (5,6). The A-site of the ribosome carries the second codon of the open reading frame (ORF) which accepts the corresponding aa-tRNA, delivered by eukaryotic elongation factor eEF1A in complex with GTP (5,6). Correct codon–anticodon base pairing triggers GTP hydrolysis by eEF1A, followed by release of the factor and accommodation of the aa-tRNA into the A-site (5). Subsequently, the P-site peptidyl-tRNA forms a peptide bond and the ratcheting movement of the ribosome triggers translocation by moving tRNAs into the hybrid P/E and A/P states. Thus, the P-site is critical for several functions during the initiation and elongation phases of translation (3–5). It should be mentioned that the A-, P- and E-sites are formed by both ribosomal RNA (rRNA) and ribosomal proteins, the later of which appear to play important roles in decoding, accommodation and stabilization of tRNAs (6).

**Figure 1.**
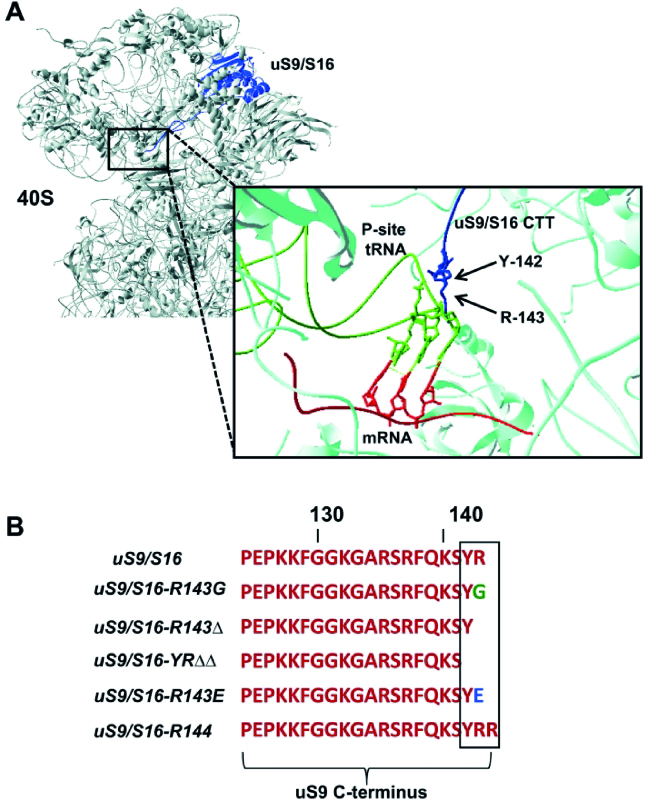
Structure and sequence analysis of uS9. (**A**) Location of ribosomal protein uS9 (formerly called S16 in yeast) in the head region of the small subunit (40S) of the eukaryotic (yeast) ribosome. The uS9 protein is depicted in navy blue and the 40S subunit is shown in grey. Inset: The CTT of uS9 is shown in navy blue with its last two amino acid residues, Tyrosine (Y) and Arginine (R), and the 40S ribosomal subunit shown in cyan. The Met-tRNA_i_^Met^ is shown in green and mRNA is shown in red. The C-terminal Tyr and Arg residues in the uS9 CTT contact the anticodon stem loop of Met-tRNA_i_^Met^ base paired with the AUG codon in the mRNA. PDB files 4V88 and 4KZZ were used for visualization using Swiss Pdbviewer. (**B**) C-terminal end sequences of the wild-type uS9 and uS9 C-terminal tail (CTT) mutants used in this study. Truncations, additions and substitutions introduced in uS9 are boxed.

We previously showed that the C-terminal tail (CTT) of the universally conserved ribosomal protein uS9 (yeast S16), which extends towards the mRNA cleft and contributes to the molecular environment of the P-site (Figure 1A), is required for efficient translation initiation and reinitiation in yeast cells (7). Moreover, this role of the CTT was mapped to the universally conserved C-terminal arginine residue (Arg-143 in yeast) and the partially variable penultimate tyrosine (Tyr-142 in yeast) (7).

The focus of the current study was to further dissect the function of the uS9/S16 (referred to herein as uS9) CTT during the initiation and elongation phases of translation. This was accomplished by generating and characterizing *Saccharomyces cerevisiae* strains expressing mutant variants of uS9 in which the length of the CTT or the charge of the last C-terminal residue was changed. During initiation, these mutants demonstrated a Gcn− phenotype with a defect in scanning and AUG recognition, as well as increased accumulation of eIF1 and eIF2 *in vivo*. In reconstituted 43S and 48S PICs, the mutants showed increased accumulation of eIF2 and compromised eIF2•GTP hydrolysis, respectively. These results suggest that the uS9 C-terminal residues are critical regulators of events surrounding 43S/48S PIC formation, including TC recruitment, scanning, and the appropriate response to the presence of an AUG start codon in the P-site. We also propose that the uS9 CTT plays an important role in ensuring the efficiency and fidelity of elongation since elimination of the last two residues of the CTT resulted in increased resistance to anisomycin (an antibiotic that prevents elongation by blocking peptide bond formation), decreased programmed ribosomal frameshifting (PRF) efficiency, and reduced polyribosomal association of eEF1A. It has been previously reported that eEF1A mutant (N153T) which displays enhanced resistance to anisomycin like drug (preussin) and decreased PRF efficiency, also exhibits stimulated intrinsic GTPase activity (8,9). Thus, we hypothesize that in addition to its role in initiation, the C-terminally conserved residues of the uS9/S16 may also ensure correct placement of eEF1A•GTP•aa-tRNA ternary complex at the decoding center, and regulated GTP hydrolysis during elongation phase of translation. Overall, these data indicate that the uS9 CTT (its length and the nature of its C-terminal residues) has evolved to control the complex interplay of events surrounding accommodation of initiator and elongator tRNAs at the P- and A-sites of the ribosome.

## MATERIALS AND METHODS

### Yeast strains and growth conditions

*uS9/S16, uS9/S16-R143G, uS9/S16-R143Δ, uS9/S16-YRΔΔ* strains (Table 1) have been previously described (7). In these strains, the chromosomal *RPS16A* and *RPS16B* genes are deleted and mutant or wild-type *RPS16* alleles are present on high-copy number plasmids. Strains *uS9/S16-R143E* and *uS9/S16-R144* were designed similarly and obtained as follows: the desired mutations were introduced into the *RPS16A* gene (expressed from the *RPS28* promoter) on high-copy plasmid K1005 (Yeplac195-pRPS28-FLAG-RPS16; URA3; 2μ; a kind gift from Dr. Philipp Milkereit, University of Regensburg, Germany) by site targeted mutagenesis using primers 5′-CCAAAAATCTTACGAATAAGAAATTGTGGGG-3′ forward and 5′-CCCCACAATTTCTTATTCGTAAGAT-TTTTGG-3′ reverse (*uS9/S16-R143E*); 5′-CCAAAAATCTTACCGTCGTTAAGAAATTGTG-3′ forward and 5′-CACAATTTCTTAACGACGGTAAGATTTTTGG-3′ reverse (*uS9/S16-R144*). Plasmids containing the desired mutations were then transformed into strain Y-318 (pGAL-RPS16A) *his3-1, leu2-0, met15-0, LYS, ura3-0, rps16B··kanMX4, rps16A::HIS3 < pGAL-RPS16A; LEU2, ARS1, CEN4>*, lacking the chromosomal genes encoding the two isoforms of *RPS16* and harboring a low-copy plasmid containing *RPS16A* under the glucose-repressible *GAL* promoter (10) (a kind gift from Dr Philipp Milkereit, University of Regensburg, Germany). The resulting strains were grown in glucose-containing medium to block expression from *pGAL-RPS16A; LEU2, ARS1, CEN4* and thus *RPS16* expressed from Yeplac195-pRPS28-FLAG-RPS16 becomes the major source of uS9/S16 protein expressed in these strains under glucose growth conditions. The strains for GCN4-LacZ and SUI5 assays were obtained as follows: K1005 vector (Yeplac195-pRPS28-FLAG-RPS16; URA3; 2μ) harboring wild-type or mutant *RPS16* was digested with PstI/NarI and the pRPS28-FLAG-RPS16 portion was inserted into PstI/ClaI-digested pRS421 (2μ, *MET15*) vector. The resulting pRS421_RPS16 constructs were transformed into Y-318 strains already harboring K1005 plasmid with the *RPS16* wild-type sequence. K1005 constructs were eliminated from the resulting strains by 5-fluoroorotic acid (5-FOA) selection, leading to generation of yeast strains expressing wild-type or mutant uS9/S16 (expressed from pRS421 plasmids). Yeast cultures were grown as indicated using either synthetic medium containing 0.67% Difco yeast nitrogen base, 1% ammonium sulfate, 2% glucose and the appropriate amino acids or YEPD medium (11). Transformation of plasmids into yeast cells was done using the lithium acetate method (12). For polysome analysis, yeast cells were grown in YEPD medium with 2% glucose. For antibiotic sensitivity assays, overnight yeast cultures were diluted to OD_600_ = 0.3 and 300 μl of the resulting suspensions were plated onto YPED plates. Five mm diameter wells were created in the center of the plates and 20 μl of 1 μg/ml anisomycin solution was applied to the wells. The plates were then incubated at 30°C for 3 days and the diameters of growth inhibition zones around the antibiotic well were measured. At least three independent assays were performed.

**Table 1.** *S. cerevisiae* strains

Strain	Genotype	Source/ reference
*uS9/S16*	*his3-1, leu2-0, met15-0, LYS, ura3-0, rps16B::kanMX4, rps16A::HIS3 <pGAL-RPS16A; LEU2, ARS1, CEN4><pRPS28-RPS16A; MET15, 2μ>*	(7)
*uS9/S16-R143G*	*his3-1, leu2-0, met15-0, LYS, ura3-0, rps16B::kanMX4, rps16A::HIS3 <pGAL-RPS16A; LEU2, ARS1, CEN4><pRPS28-RPS16A-R143G; MET15, 2μ>*	(7)
*uS9/S16-R143Δ*	*his3-1, leu2-0, met15-0, LYS, ura3-0, rps16B::kanMX4, rps16A::HIS3 <pGAL-RPS16A; LEU2, ARS1, CEN4><pRPS28-RPS16A-R143Δ; MET15, 2μ>*	(7)
*uS9/S16-YRΔΔ*	*his3-1, leu2-0, met15-0, LYS, ura3-0, rps16B::kanMX4, rps16A::HIS3 <pGAL-RPS16A; LEU2, ARS1, CEN4> <pRPS28-RPS16A-Y142ΔR143Δ; MET15, 2μ>*	(7)
*uS9/S16-R143E*	*his3-1, leu2-0, met15-0, LYS, ura3-0, rps16B::kanMX4, rps16A::HIS3 <pGAL-RPS16A; LEU2, ARS1, CEN4><pRPS28-RPS16A-R143E; MET15, 2μ>*	This work
*uS9/S16-R144*	*his3-1, leu2-0, met15-0, LYS, ura3-0, rps16B::kanMX4, rps16A::HIS3 <pGAL-RPS16A; LEU2, ARS1, CEN4><pRPS28-RPS16A-R144; MET15, 2μ>*	This work

### Reporter plasmids

Yeast p180, pM226 and pM199 reporter plasmids (13,14), derivatives of the YCp50 (CEN, URA3) vector bearing *GCN4-lacZ* alleles, were kindly provided by Drs. Thomas Dever and Alan Hinnebusch (National Institutes of Health). β-galactosidase activity was measured under normal (without 3-Amino-1,2,4-triazole (3-AT)) and/or amino acid starved (+3AT) conditions and compared between mutant strains and the wild-type. When 3AT was used a pairwise comparison (mutant strain (-3AT) vs wild-type (-3AT) and mutant strain (+3AT) vs wild-type (+3AT)) was done in each respective case. The YCp *SUI5-G31R* plasmid (derivative of YCplac33 (CEN, URA3)) harboring the *TIF5-G31R* allele (15) was a kind gift from Dr. Leos Valasek (Institute of Microbiology, Academy of Sciences of the Czech Republic). *HIS4-LacZ* reporter constructs with AUG or UUG initiation codons (p367 and p391, respectively; kindly provided by Dr Alan Hinnebusch, National Institutes of Health) were used for assaying the efficiency of start codon recognition/Sui phenotypes (16). Plasmids were transformed into wild-type and mutant strains and grown on minimal YNB medium. Programmed –1 and +1 frameshifting test reporters containing L-A (pJD376), Ty1 (pJD377) or Ty3 (pJD379) frameshift signals between the Renilla and firefly luciferase genes (17,18) were provided by Dr Jonathan D. Dinman (University of Maryland). All luciferase reporter plasmids were transformed into wild-type and mutant strains and grown on minimal YNB medium.

### Fractionation of polyribosomes

Fractionation of polyribosomes was done essentially as described before (19,20) using 10–50% and/or 10–30% sucrose gradients and centrifugation at 17 000 and 20 000 rpm, respectively, for 18 h using a Beckman SW32.1 rotor. All procedures were performed at 4°C. Yeast cells from 50 ml of log phase culture were pelleted, treated for 10 min with 100 μg/ml cycloheximide and re-pelleted. Cell extracts were made by glass bead cell disruption (3–5 cycles of 1 min each), with intermittent cooling on ice. The following buffer was used: 20 mM HEPES–KOH, pH 7.4, containing 100 mM KCl, 2.5 mM magnesium acetate, 14.4 mM β-mercaptoethanol, 100 μg/ ml cycloheximide. Cell debris was removed by centrifugation at 7000 rpm (4500 × g) for 8 min. Polyribosomes were resolved by sucrose density gradient centrifugation (15 ml total volume) by loading 26 OD_260_ units of cell extracts. Gradients were collected using the ISCO Programmable Density Gradient System with continuous monitoring at 254 nm using an ISCO UA-6 absorbance detector. Determination of the ratios of 80S monosomes to polyribosomes was done essentially as described before (20). Fractionation of cell extracts using formaldehyde cross-linking was done as described by Nielsen *et al.* (14). For western blotting, proteins collected from sucrose gradient fractions were solubilized in sodium dodecyl sulfate (SDS) polyacrylamide gel electrophoresis (PAGE) sample buffer for 10 min at 95°C, chilled on ice for 5 min and loaded onto polyacrylamide gels.

### rRNA analysis

Yeast strains were grown at 30°C in complete medium to mid-logarithmic phase. rRNA was extracted and subject to denaturing gel electrophoresis as described (20). Gels were stained with ethidium bromide and scanned using a Typhoon imaging scanner.

### Western blotting

Western blotting was done following standard procedures (21). Anti-rpS5 antibodies against the C-terminal conserved rpS5 peptide AIKKKDELERVAKSNRC were described previously (20). Anti-eIF2α (22) and anti-eIF1 (23) were kindly provided by Drs. Thomas Dever and Alan Hinnebusch (National Institutes of Health). Anti-eEF1A antibodies (ED7001) were obtained from Kerafast. Goat anti-rabbit HRP-conjugated antibodies and an enhanced chemiluminescence detection kit (ECLTM, GE Healthcare, Piscataway, NJ, USA) were used for detection.

### β-Galactosidase assays

For GCN4-lacZ assays, cells were grown for 2 h in minimal synthetic (SD) medium supplemented with appropriate amino acids containing 2% glucose. To invoke amino acid starvation, 3-amino-1,2,4-triazole (3-AT) (final concentration 30 mM) was then added and the incubation was continued for additional 5 h. Cells were harvested, and extracts were prepared by subsequent cycles of cell freezing in liquid nitrogen and thawing at 37°C. For assaying Sui phenotypes, cells were grown in minimal synthetic medium and β-galactosidase activity was assayed in whole cell extracts. β-Galactosidase activity was measured following the protocol described in the Clontech Yeast Protocols Handbook using *O*-nitrophenyl-β-d-galactopyranoside as a substrate. Two sided *P*-values were calculated (Student's *t*-test).

### GTPase assay

pTYB2 expression vectors harboring genes for eIFs1, 1A and 5 were obtained from Addgene and the respective proteins were purified as described in (24,25). His-tagged eIF2 was purified from yeast strain GP3511 and 40S yeast ribosomal subunits were isolated as described in (26). The model mRNA template and tRNA_i_^Met^ were purchased from IDT and tRNA probes, respectively. Manually quenched GTPase experiments were conducted as follows: TC was prepared at 2× concentration by mixing 1× reaction buffer (30 mM HEPES–KOH, pH 7.4, 100 mM KOAc, 3 mM Mg(OAc)_2_, 2 mM DTT) with 1.6 μM eIF2, 1.6 μM Met-tRNA_i_ and 125 pM GTPγ[^32^P] and incubating the mixture for 5 min at 26°C. Ribosomal complexes were prepared at 2× concentration by mixing 400 nM 40S ribosomal subunits, 1.6 μM (each) eIF1, eIF1A and eIF5, 2 μM model mRNA and 2 mM GDP disodium salt. For each time point, 2 μl of TC was mixed with 2 μl of ribosomal complex for the desired time, after which 2 μl was removed and quenched into 6 μl of quench/dye solution (90% formamide, 0.02% bromophenol blue and 100 mM of EDTA). To quantify the extent of GTP hydrolysis, 15% polyacrylamide TBE gels were run to separate GTPγ[^32^P] from free ^32^P_i_ followed by PhoshorImager analysis.

### Miscellaneous

Molecular cloning was performed following standard procedures. Deoxyribonucleic acid (DNA) sequencing was accomplished by the DNA Sequencing Core facility at Cleveland Clinic. Sodium dodecyl sulfate polyacrylamide gel electrophoresis (SDS-PAGE) was performed according to Laemmli (27). Luciferase activity was measured using a dual luciferase assay kit (Promega Madison, WI, USA) as described by Dinman and co-authors (17,18,28). Two sided *P*-values were calculated (Student's *t*-test).

## RESULTS

We previously demonstrated that yeast uS9 mutants lacking the last two C-terminal residues of the CTT or containing a neutral Gly in place of the last C-terminal Arg of the CTT displayed a slow-growth (Slg−) phenotype, reduced rate of bulk translation initiation, impaired derepression of GCN4 mRNA translation (Gcn− phenotype) and accumulation of eIF1 and eIF2 on native 40S subunits (7). The Slg− phenotype was suppressed in these strains (7) by introducing an eIF5 mutant (G31R) with elevated GTPase-activating protein (GAP) function (29). Based on these results, we proposed that the uS9 CTT influences initiation events surrounding recruitment of the eIF2•GTP•Met-tRNA_i_^Met^ ternary complex (TC) and promotes eIF5-stimulated GTP-hydrolysis or Pi release (7). It was not clear, however, to what extent the exact position of the uS9 CTT within the ribosome and the translation complex (determined in part by its length) and/or the nature of the last amino acids of the CTT are important for these events. The experiments described below addressed these questions as well as the role of the uS9 CTT in elongation.

### The length and charge of the uS9 CTT are critically important for efficient translation initiation

**Figure 2.**
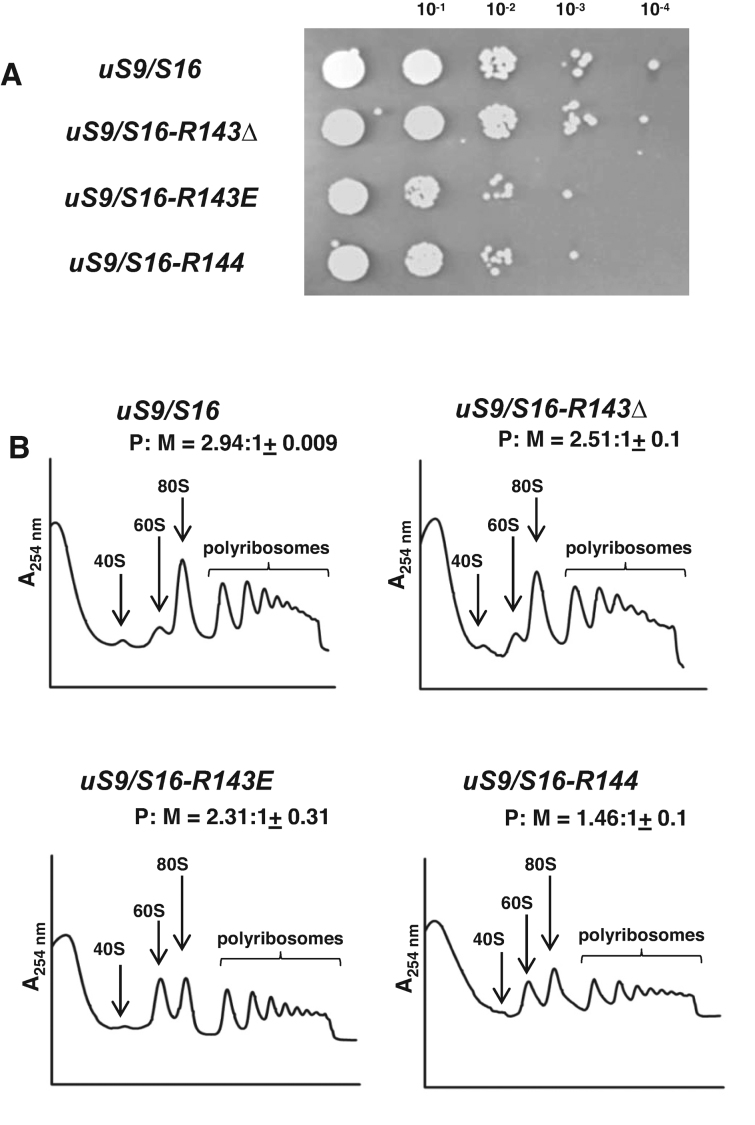
The uS9 C-terminal tail is essential for translation initiation in yeast cells. (**A**) Growth of wild-type and uS9 CTT mutant yeast strains (*uS9/S16 R143Δ, uS9/S16 R143E, uS9/S16 R144*). Cells were grown for 36 h on solid YEPD agar medium containing 2% glucose. (**B**) Translation initiation defects in uS9 mutants. Ribosome profiles of wild-type and mutant yeast strains. Whole cell extracts of the yeast strains were resolved by velocity sedimentation through 10–50% sucrose gradients. Fractions were collected while scanning at A254. The positions of different ribosomal species are indicated. Ratios of the area under the polyribosomal (P) and 80S (monosomal; M) peaks are shown (P:M) (mean ± standard error of the mean [SEM]).

CryoEM and X-ray structures of 40S ribosomal subunits complexed with tRNA and mRNA (30,31) showed that the conserved C-terminal residue of the uS9 CTT (a positively charged Arg) contacts the initiator tRNA when it is base-paired to the AUG codon in the P-site (Figure 1A). To investigate the functional significance of the charge and length of the uS9 CTT, we analyzed a set of *S. cerevisiae* mutants (Figure 1B). In addition to using several mutants described previously, namely *uS9/S16-R143G, uS9/S16-R143Δ* and *uS9/S16-YRΔΔ* (7), we produced two new strains in which we either replaced the C-terminal arginine of uS9 (Arg-143) with a negatively charged glutamic acid (mutant *uS9/S16-R143E*) or extended the uS9 C-terminal end by one additional Arg residue (mutant *uS9/S16-R144*) (Figure 1B). Both of these mutants displayed a slow growth phenotype (Figure 2A) that was not as severe as that previously observed for the *uS9/S16-YRΔΔ* strain (7), yet more pronounced than that of the *uS9/S16-R143Δ* strain. We next tested whether the Slg− phenotypes of the *R143E* and *R144* strains were associated with a general defect in translation initiation by analyzing polyribosomes prepared by sucrose density gradient centrifugation of cytoplasmic extracts from the *uS9/S16* (WT), *uS9/S16-R143E* and *uS9/S16-R144* strains. This revealed a reduced polyribosome (P) to monosome (80S) ratio (P:M) in *uS9/S16-R143Δ, uS9/S16-R143E* and *uS9/S16-R144* strains as compared to the wild-type strain (Figure 2B). Interestingly, the reduction was more severe in the *uS9/S16-R144* strain (P:M = 1.46) harboring an extra Arg at the C-terminus of the uS9 CTT than in strains *uS9/S16-R143Δ* (P:M = 2.51) and *uS9/S16-R143E* (P:M = 2.31) (Figure 2B). Since a reduced P:M ratio is a characteristic phenotype of mutations that impair translation initiation, these data led us to hypothesize that the length of the uS9 CTT may be a more important determinant of efficient translation initiation than the charge of the C-terminal residue per se. Nevertheless, the charge of this residue appears to play an important role as well. Interestingly, in our sucrose density gradient centrifugation experiments, both *uS9/S16-R143E* and *uS9/S16-R144* strains also showed an increased peak for 60S subunits, suggesting a possible defect in subunit joining (32,33) and/or ribosome biogenesis. This defect was not observed in strain *uS9/S16-R143Δ*, nor in previously described *uS9/S16-R143G* and *uS9/S16-YRΔΔ* strains (7). Analysis of rRNA under denaturing conditions indicated that ribosome biogenesis is not severely affected in the *uS9/S16-R143E* and *uS9/S16-R144* strains since their 18S/25S ratios were comparable to those in the wild-type strain (Supplementary Figure S1). Therefore, a defect in subunit joining is most likely the main factor underlying the increased 60S peak in these mutants.

### Translation reinitiation is compromised in uS9 mutants

To gain further insight into the role of the uS9 CTT in translation initiation, we used reporter constructs under the control of *GCN4* regulatory elements as sensitive indicators of the rate of TC binding to 40S ribosomes *in vivo* (34). Regulation of *GCN4* translation is exerted via a reinitiation process involving four small upstream open reading frames (uORFs) preceding the *GCN4* ORF (34). Following translation of the 5′ proximal uORF (uORF1), reinitiation depends on *de novo* recruitment of the eIF2 TC, which is required to recognize the next AUG codon, and is thus exquisitely sensitive to eIF2•GTP levels (for review, see (34)).

**Figure 3.**
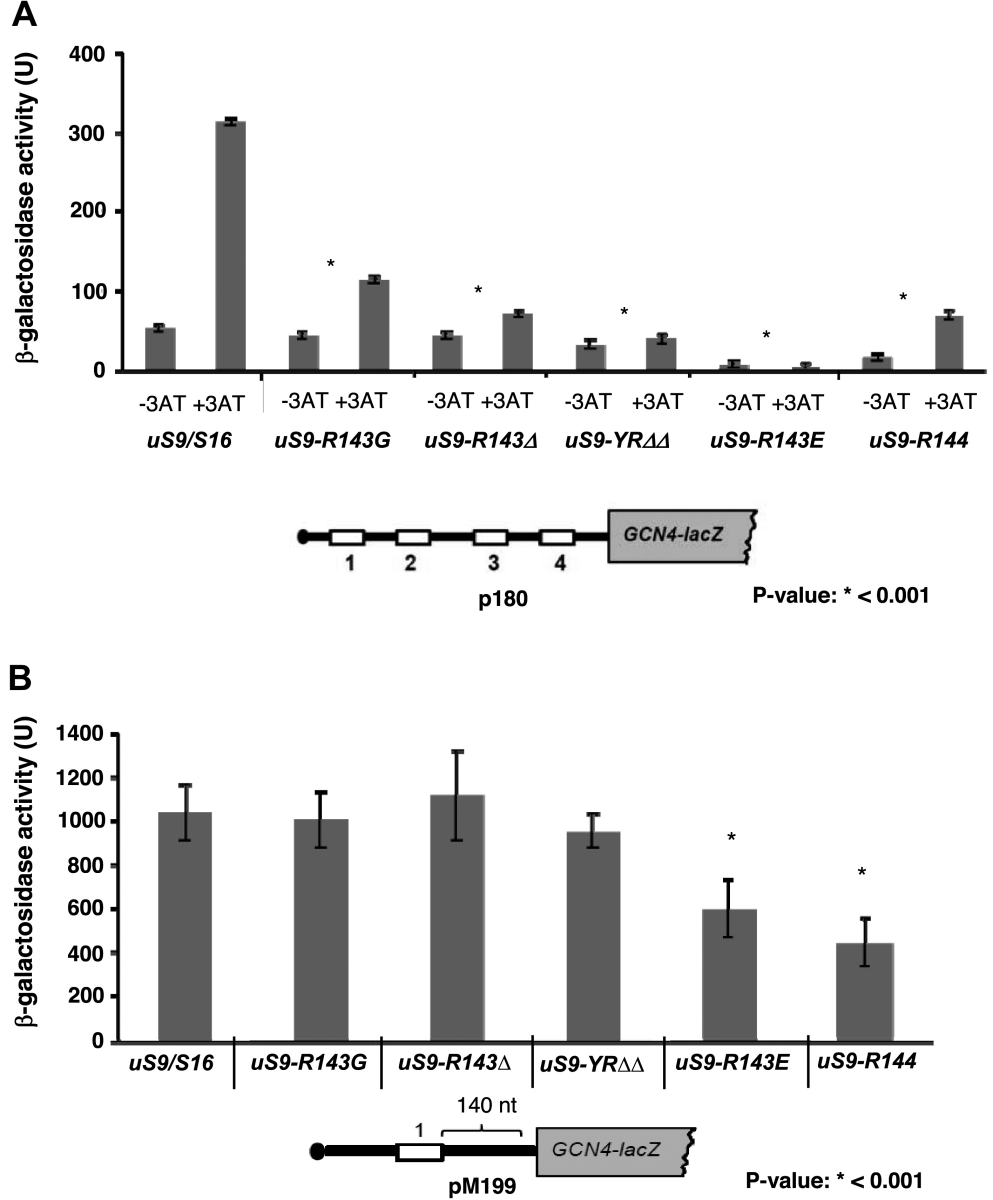
Changes in the length and charge of the uS9 CTT cause defects in translation reinitiation and resumption of scanning during *GCN4* translation. (**A**) Translation re-initiation defects in uS9 mutants. Wild-type and mutant yeast strains were transformed with p180 *GCN4-lacZ* reporter construct and assayed for GCN4 re-initiation efficiency using 3-AT. p180 contains the wild-type GCN4 mRNA leader (all four uORFs). β-galactosidase activity (units) were measured under normal (without 3AT) and amino acid starved (+3AT) conditions. (**B**) *GCN4-lacZ* reporter activity. Wild-type and mutant yeast strains (*uS9/S16-R143G, uS9/S16-R143Δ, uS9/S16-YRΔΔ, uS9/S16-R143E, uS9/S16-R144*) were transformed with pM199 containing only uORF1, which is 140 nucleotides away from the *GCN4* ORF. β-Galactosidase activity (units) was measured under normal conditions (-3AT) and is shown as the mean ±SEM from three biological replicates of three technical replicates each.

**Figure 4.**
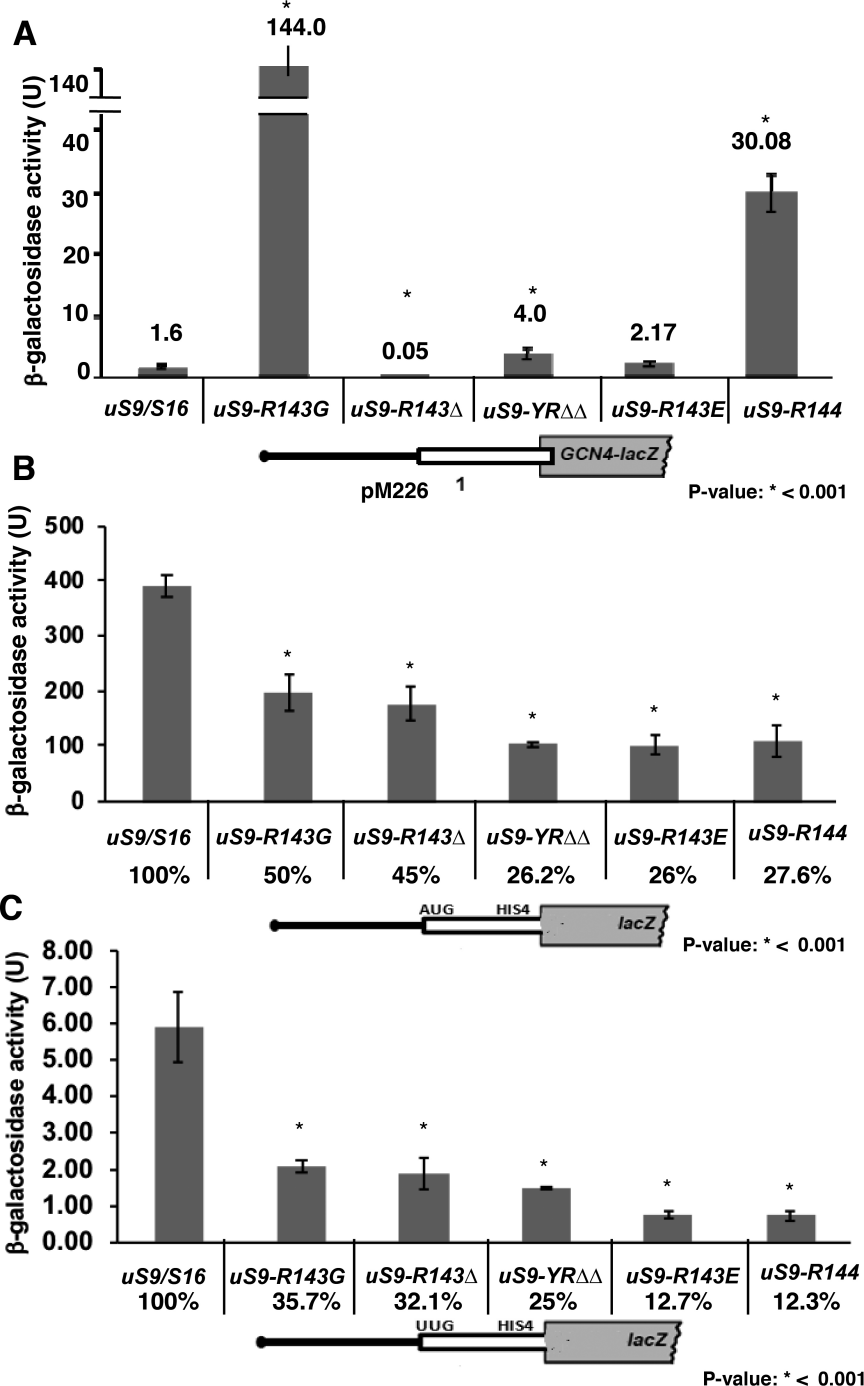
Changes in the length and charge of the uS9 CTT result in a leaky scanning phenotype as well as compromised AUG and UUG codon recognition. (**A**) *GCN4-lacZ* reporter activity in wild-type and mutant yeast strains (*uS9/S16-R143G, uS9/S16-R143Δ, uS9/S16-YRΔΔ, uS9/S16-R143E, uS9/S16-R144*) transformed with pM226 containing uORF1 extended into the GCN4 ORF. (**B**) Activity of *HIS4-LacZ* reporter constructs harboring AUG (B) or UUG (**C**) initiation codons following transformation into wild-type and mutant yeast strains. Mean β-galactosidase activity ± SEM determined from three biological replicates of three technical replicates each is shown.

To assess reinitiation in yeast strains expressing C-terminally modified versions of uS9, we transformed a set of *GCN4-lacZ* reporters (Figures 3 and 4 and Supplementary Figures S2 and S3) into wild-type strain *uS9/S16* and its isogenic derivatives *uS9/S16-R143G, uS9/S16-R143Δ, uS9/S16-YRΔΔ, uS9/S16-R143E* and *uS9/S16-R144*. To invoke amino acid starvation, cells were treated with 3-amino-1,2,4-triazole (3-AT), an inhibitor of histidine biosynthesis. Measurement of *GCN4-lacZ* reporter expression from the p180 reporter plasmid containing all four *GCN4* uORFs showed that the tested uS9 CTT truncations, mutations and extensions all substantially reduced translation reinitiation (Figure 3A and Supplementary Figure S2). Compared to the wild-type strain, *GCN4-lacZ* expression from the p180 construct was reduced by ∼3-fold in strain *uS9/S16-R143G*, ∼4-fold in strain *uS9/S16-R143Δ*, ∼8-fold in strain *uS9/S16-YRΔΔ*, ∼40-fold in strain *uS9/S16-R143E* and ∼4-fold in strain *uS9/S16-R144*. Notably, substitution of the uS9 C-terminal Arg with Glu (in strain *uS9/S16-R143E*) resulted in both near complete abrogation of reinitiation induction and a reduced basal level of reinitiation. Cell growth assays also revealed increased sensitivity of *uS9/S16-R143E* and *uS9/S16-R144* strains to 3-AT (Supplementary Figure S4, +3AT). These observations led us to conclude that the tested uS9 CTT mutants exhibit a strong ‘general control non-derepressible’ (Gcn−) phenotype (34). Based on this, we embarked on a more thorough analysis of the role of the uS9 CTT in translation initiation and reinitiation, and investigation of the mechanism underlying the Gcn− phenotype seen in the mutants.

### uS9 CTT mutants display defects in resumption of scanning during GCN4 translation *in vivo*

During the process of *GCN4* mRNA translation initiation, uORF1 translation is followed by a reinitiation (REI)-specific phase followed by a general translation initiation-like phase. The REI-specific phase relies mainly on the ability of the 40S ribosomal subunits to remain attached to the mRNA after uORF1 translation termination. This is followed by recruitment of factors involved in resumption of ribosomal scanning on the same mRNA (35). To evaluate the effect of uS9 CTT mutations on resumption of scanning after uORF1 translation, we assayed a *GCN4-lacZ* reporter containing uORF1 positioned 140 nt from the *GCN4* AUG codon as the only uORF in the leader (construct pM199; Figure 3B and Supplementary Figure S3). A failure to resume scanning following uORF1 translation is expected to lead to reduced expression of the pM199 construct. While *uS9/S16-R143G, uS9/S16-R143Δ* and *uS9/S16-YRΔΔ* strains did not show any significant change in β-galactosidase expression from this construct compared to the wild-type strain, *uS9/S16-R143E* and *uS9/S16-R144* strains showed significant decreases of ∼50–60% (Figure 3B and Supplementary Figure S3). Thus a change in charge at the uS9 C-terminus (from a positive Arg to a negative Glu in *uS9/S16-R143E*) or an increase in charge and length (*uS9/S16-R144*) critically affects either the recruitment of ribosomes to the mRNA and/or the scanning process. Specifically, this could be due to failure of the 40S subunits to remain attached to the GCN4 mRNA and/or impairment of their ability to acquire scanning promoting factors (eIF1 and eIF1A) (35–37). Delayed TC recruitment is another possible explanation for the observed effect of these uS9 mutations on scanning. It should also be noted that the degree of the defect in resumption of scanning on the pM199 reporter construct in *uS9/S16-R143E* (∼2-fold reduced vs. wild-type) cannot alone account for its strong Gcn− phenotype (∼44-fold reduced versus wild-type) (Figure 3A and Figure S2). This is consistent with the finding that *GCN4-lacZ* expression from pM199 was not significantly altered in *uS9/S16-R143G, uS9/S16-R143Δ* and *uS9/S16-YRΔΔ* strains although they also displayed a Gcn− phenotype.

### uS9 C-terminal residues promote accurate cognate and non-cognate codon recognition

Since the last two C-terminal residues of uS9 interact with the initiator tRNA base paired to the AUG codon at the ribosomal P-site (30,31 and Figure 1A), we were interested in determining whether these residues are indeed important for recognition of the start codon during scanning. uS9 mutants exhibit a Gcn− phenotype (Figure 3A and Figure S2) and a possible defect underlying this phenotype could be the failure of scanning 40S subunits to recognize the uORF1 AUG codon (leaky scanning), with initiation at non-permissive uORFs 2–4 instead. To test this possibility, we assayed *GCN4-lacZ* expression in whole-cell extracts (WCEs) from wild-type and uS9 CTT mutant strains harboring reporter construct pM226. This construct carries a solitary uORF1 that is extended to overlap the *GCN4-lacZ* coding region, which destroys the ability of ribosomes to reinitiate at *GCN4* after terminating at the elongated uORF1 stop codon (13). Under these circumstances, *GCN4* can be translated only by ribosomes that fail to recognize the AUG codon in uORF1. *uS9/S16-R143G* and *uS9/S16-R144* mutant strains displayed considerably higher levels of pM226 reporter expression than the wild-type strain (∼90- and 20-fold, respectively; Figure 4A and Supplementary Figure S3). Thus, these mutants exhibit a strong leaky scanning defect, which may account for their Gcn− phenotype (Figure 3A and Supplementary Figure S2). Since uORF1 acts as a strong positive regulator of translation initiation, the derepression defect in these mutants is exerted by allowing a fraction of PICs, scanning from the cap, to bypass this uORF. For *uS9/S16-YRΔΔ*, a moderate ∼3-fold increase in *GCN4-lacZ* expression from pM226 was observed (Figure 4A and Supplementary Figure S3); this would be only a minor contributor to its notably reduced expression from the p180 reporter construct (∼8-fold) (Figure 3A and Supplementary Figure S2). Likewise, *uS9/S16-R143Δ* and *uS9/S16-R143E* strains showed minimal *GCN4* expression, deducing that only a negligible amount of 40S ribosomes leaky scan the uORF1 AUG from these mutants, like in wild-type (Figure 4A and Supplementary Figure S3).

To confirm the effects of uS9 CTT modifications on the stringency of start codon selection during scanning, we measured the expression of *HIS4-lacZ* reporters containing either AUG or UUG as start codons. Remarkably, uS9 CTT mutations reduced initiation at both AUG and UUG codons. AUG recognition in *uS9/S16-R143G* and *uS9/S16-R143Δ* strains was reduced to ∼50% and 45% of that in the wild-type strain, respectively, and in *uS9/S16-YRΔΔ, uS9/S16-R143E* and *uS9/S16-R144* strains, it was further reduced to ∼26–27% of wild-type (Figure 4B and Supplementary Figure S3). These data corroborate the leaky scanning defect described above. Therefore, we propose that compromised AUG recognition (in uORF1) is likely responsible for the Gcn− phenotype of uS9 mutants. Moreover, UUG codon selection was found to be even more dramatically affected than AUG recognition in uS9 CTT mutant strains (Figure 4C and Supplementary Figure S3). This observation further supports a role for the uS9 CTT in start codon selection, as a more severe defect in recognition of UUG versus AUG in uS9 CTT mutant strains would have been exacerbated by the preexisting base pairing mismatch between the UUG codon and the anticodon of tRNA_i_. The highly compromised cognate/non-cognate codon recognition rates (along with the leaky scanning defect) in uS9 mutants suggests that the C-terminal Arg and penultimate Tyr of the uS9 CTT are required for efficient start codon selection at the P-site during translation initiation.

### uS9 C-terminal tail mutant strains exhibit altered association of eIF1 and eIF2α with 48S complexes

**Figure 5.**
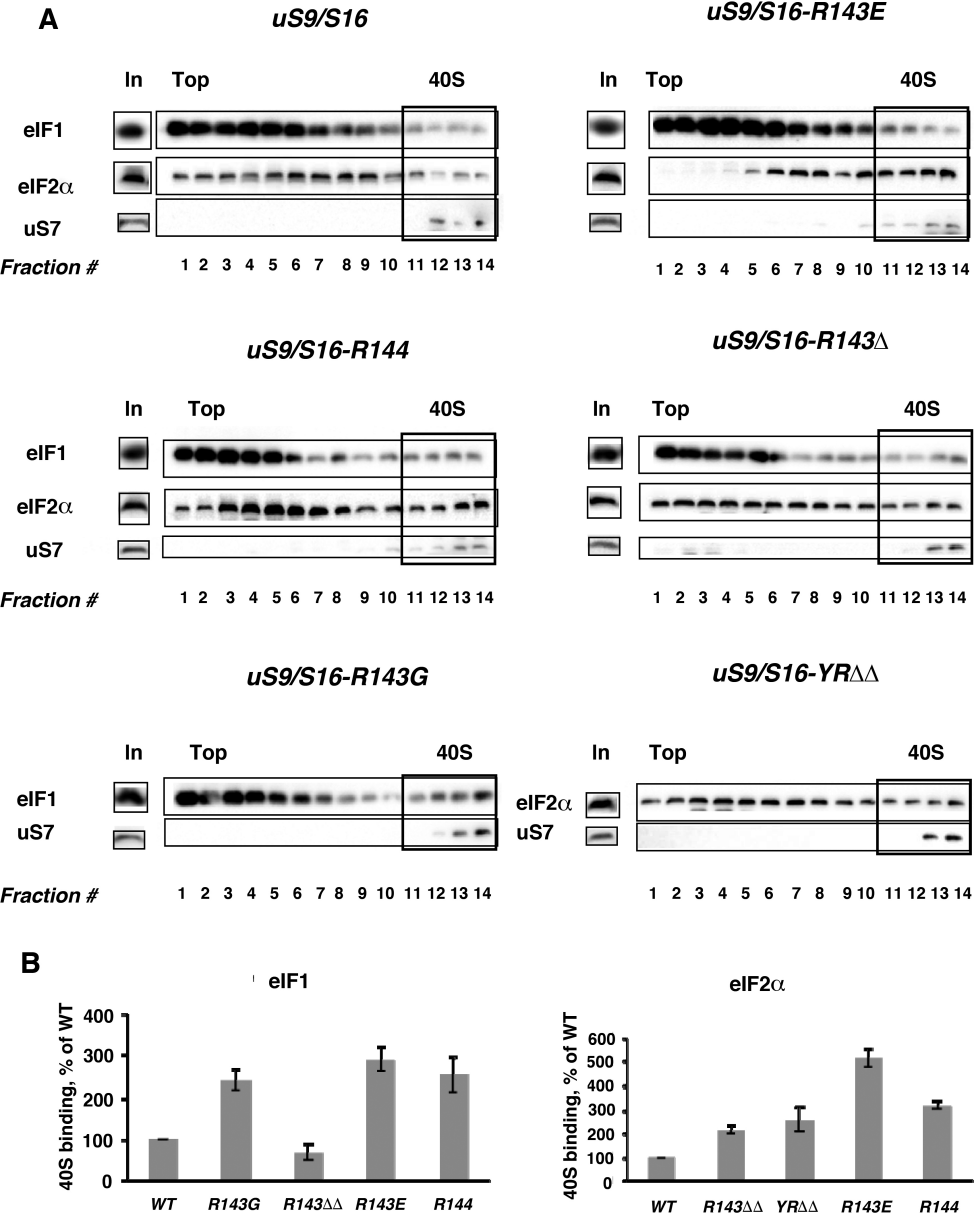
Association of eIF1 and eIF2α with 40S ribosomal subunits in wild-type and mutant (*uS9/S16-R143G, uS9/S16-R143Δ, uS9/S16-YRΔΔ, uS9/S16-R143E, uS9/S16-R144)* yeast strains. Extracts from isogenic wild-type and mutant strains were resolved by sucrose density gradient (10–30%) sedimentation. (**A**) Western blot analyses were performed using antibodies against eIF1, eIF2α and the ribosomal protein uS7/S5. Lanes marked ‘In’ for input contained a 7% portion of each gradient fraction. Analysis of eIF1 and eIF2α association was done using whole cell extract cross-linking with formaldehyde. (**B**) Association of eIF1 and eIF2 with 40S subunits was quantified and expressed as a percentage of 40S binding normalized against uS7.

Several eukaryotic initiation factors comprise the multifactor complex (MFC) that stimulates various steps in assembly of the 48S pre-initiation complex (PIC) (3,4). eIF1 ensures accurate start codon recognition by blocking Pi release from eIF2•GDP•Pi and stabilizing an open, scanning-competent conformation of the 40S ribosomal subunits at non-AUG codons. Recognition of the start codon at the P-site triggers dissociation of eIF1, complete hydrolysis of GTP, and displacement of Pi and eIF2•GDP from 40S subunits, thus forming a stable 48S PIC (3,31,37). eIF2 is a heterotrimer (composed of α, β and γ subunits) that binds initiator Met-tRNA_i_^Met^ to the 40S subunit in a ternary complex, aided by the MFC (3). To assess the role of the uS9 CTT in interaction of 40S subunits with MFC components (particularly eIF1 and eIF2) to form 43S/48S PICs, we used formaldehyde crosslinking to assess such interactions in vivo in wild-type and uS9 CTT mutant strains. After formaldehyde treatment, WCEs were resolved by sedimentation through sucrose density gradients and the fractions were then analyzed by western blotting using antibodies against eIF1, the eIF2α subunit of eIF2, and the 40S ribosomal subunit protein uS7. Western blot signals from fractions containing 43/48S PICs were quantified and normalized to uS7 levels. While *uS9/S16-R143G, uS9/S16-R143E* and *uS9/S16-R144* strains showed ∼2.5–3.0-fold increased association of eIF1 in 40S-containing fractions as compared to the wild-type strain, eIF1–40S binding in *uS9/S16-R143Δ* was slightly reduced (Figure 5A and B). Similarly, we observed increased association of eIF2α with 40S subunits in all tested uS9 mutant strains. The increase in eIF2–40S association seen in *uS9/S16-R143Δ, uS9/S16-YRΔΔ* and *uS9/S16-R144* (∼2–3.0-fold) was smaller than that in *uS9/S16-R143E* (∼5.0-fold) (Figure 5A and B). The increased binding of eIF1 and eIF2α to 40S subunits seen in the uS9 mutants is consistent with a disruption in the release of eIF1 and eIF2•GDP from the 48S PIC. This could be due to failure of a primary translation initiation event; for example, inadequate formation of scanning competent 43/48S PICs or defective start codon recognition (see above). Additionally, inefficient GTP hydrolysis or Pi release after encountering the AUG start codon could also explain reduced eIF2•GDP and eIF1 dissociation from the 40S subunit.

### The conserved CTT of uS9 is essential for eIF5-stimulated GTP hydrolysis

**Figure 6.**
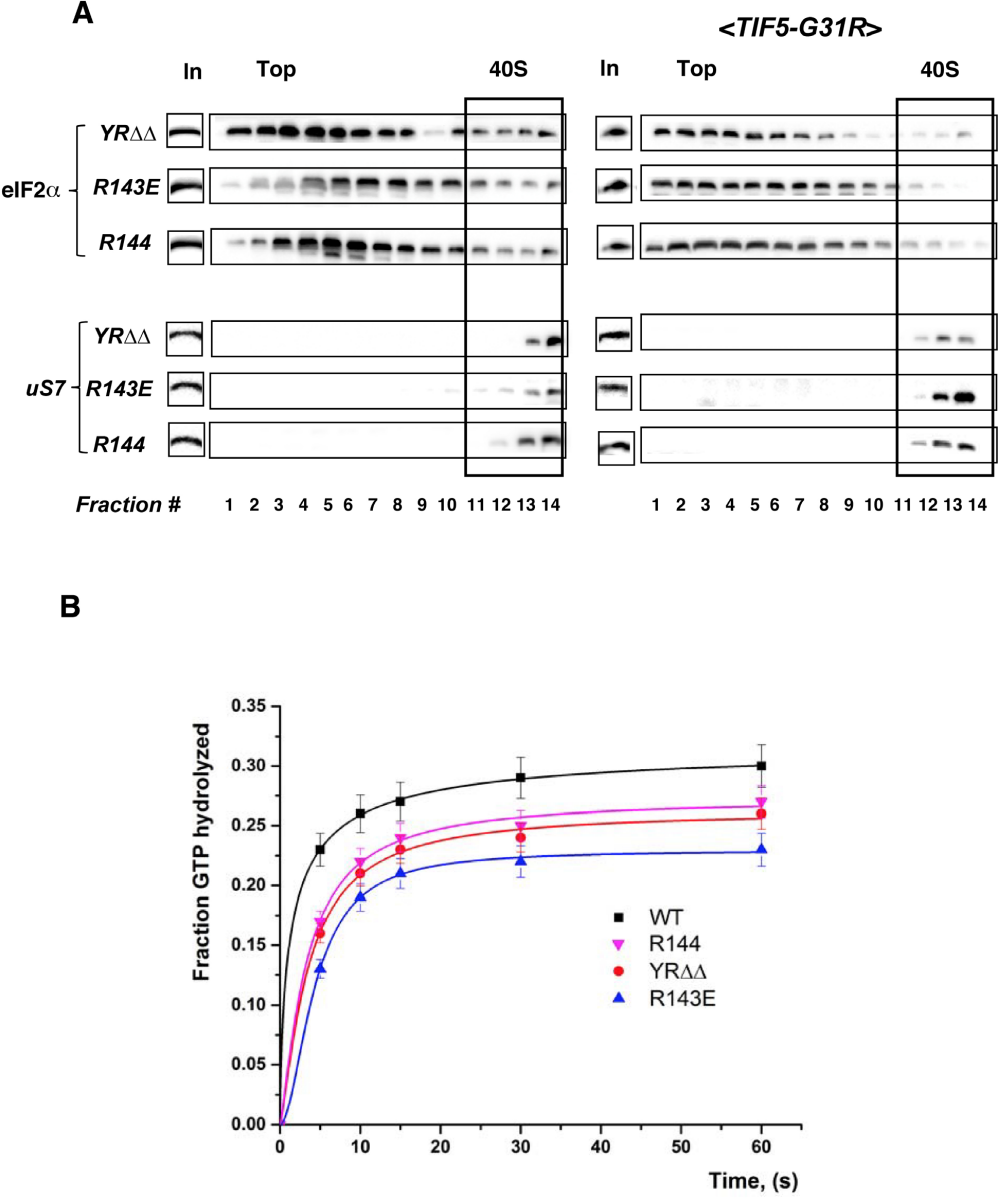
The uS9/S16 C terminal region is important for eIF5-stimulated GTP hydrolysis. (**A**) Introduction of the *TIF5-G31R* allele reverses accumulation of eIF2α on uS9 mutant 40S ribosomal subunits. Association of initiation factor eIF2α with 40S subunits in *uS9/S16-YRΔΔ, uS9/S16-R143E, uS9/S16-R144* (left panel) and *uS9/S16-YRΔΔ* <*TIF5-G31R*>, *uS9/S16-R143E*<*TIF5-G31R*>, *uS9/S16-R144* <*TIF5-G31R*> (right panel) yeast strains. Western blot analysis of individual fractions with antibodies against eIF2 and uS7 is shown. ‘In’ for input - represents a 7% portion of each gradient fraction. (**B**) GTP hydrolysis by eIF2 with wild-type and mutant yeast 40S subunits. 40S•eIF1•eIF1A•mRNA (AUG) complexes were assembled in the presence of eIF5 and mixed with TC to initiate the GTP hydrolysis reaction.

Hydrolysis of GTP-bound to the eIF2-ternary complex is mediated by eIF5 and proceeds to completion only when P_i_ is released from eIF2 after start codon recognition. After the GTP hydrolysis step, eIF1 and eIF2•GDP become dissociated from the 40S subunits, thus committing the 43S PIC to begin translation at the selected codon (3). We reasoned that if the uS9 CTT has a role in eIF5-stimulated GTP hydrolysis in the scanning complex prior to AUG recognition, the accumulation of initiation factors like eIF2 and eIF1 on 40S subunits in uS9 mutants might be due to compromised GTP hydrolysis. To test this, we first utilized the *SUI5* mutant allele eIF5-G31R, which has greater than wild type GAP function of eIF5 (29). We expected that introducing this plasmid-borne *SUI5* allele into our uS9 CTT mutant yeast strains would suppress their defective phenotypes. Indeed, in our previous study, we observed that introduction of *SUI5* eliminated the Slg− phenotype of strain *uS9/S16-YRΔΔ* (7). Here, expression of eIF5-G31R in strains *uS9/S16-R143E* and *uS9/S16-R144* reversed eIF2 accumulation on 40S subunits (Figure 6A). These results support the possibility of either delayed eIF2-bound GTP hydrolysis or defective P_i_ release from eIF2•GDP•Pi in 48S PICs during translation initiation in the uS9 CTT mutants.

To obtain further evidence that the uS9 CTT plays a role in GTP hydrolysis, we used a reconstituted translation initiation system to measure eIF5-stimulated GTP hydrolysis activity of eIF2 (24,25). *uS9/S16-YRΔΔ, uS9/S16-R143E* and *uS9/S16-R144* strains were used in these experiments since they displayed the strongest phenotypic defects (Slg−, Gcn− and a reduced rate of bulk translation initiation; Figure 2 and Figure S4, +3AT) among our panel of uS9 mutant strains. PICs were preassembled containing eIF1, eIF1A, eIF5 and a model mRNA with AUG as the start codon (as described (24)). TC assembled with GTPγ[^32^P] was added to the PICs to initiate the reaction and aliquots were quenched at different time points using EDTA. GTPase activity was monitored by measuring conversion of GTPγ[^32^P] into GDP and ^32^P_i_ (separated by 15% PAGE and analyzed by phosphorimager). It should be noted that the GTP hydrolysis reaction assayed in this manner corresponds to the fast phase with cleavage of GTP to produce GDP and P_i_. Our data showed that PICs assembled with wild-type uS9 hydrolyzed GTP with a rate constant of 14.0 × 10^−3^ s^−1^ (Figure 6B, black curve). Introduction of additional Arg residue at the uS9 C-terminus (*uS9/S16-R144*) or deletion of its last two residues (*uS9/S16-YRΔΔ*) decreased the rate constant for GTP hydrolysis to 8.4 × 10^−3^ s^−1^ (Figure 6B, pink curve) and 8.2 × 10^−3^ s^−1^ (Figure 6B, red curve), respectively. Substitution of the terminal arginine with a negatively charged glutamate (*uS9/S16-R143E*) further decreased the rate constant to 5.6 × 10^−3^ s^−1^ (Figure 6B, blue curve). Thus, altering the length or the charge of the uS9 CTT adversely affected the rate of GTP hydrolysis during translation initiation.

### uS9 CTT residues play a critical role in translation elongation

Since the uS9 CTT occupies the P-site, we hypothesized that it might be involved in translation elongation as well as initiation. One important aspect of elongation is the maintenance of translational fidelity, which ensures the production of full-length, functional proteins. To test whether changes in the uS9 CTT affect translation fidelity, we used a bicistronic dual-luciferase reporter construct containing frameshift signals between the Renilla and firefly luciferase genes such that firefly luciferase can only be produced in the event of a frameshift (17,18). Ty1 and Ty3 retrotransposon-derived frameshift signals produce +1 frameshifting, while L-A virus-derived signals produce –1 frameshifting.

**Figure 7.**
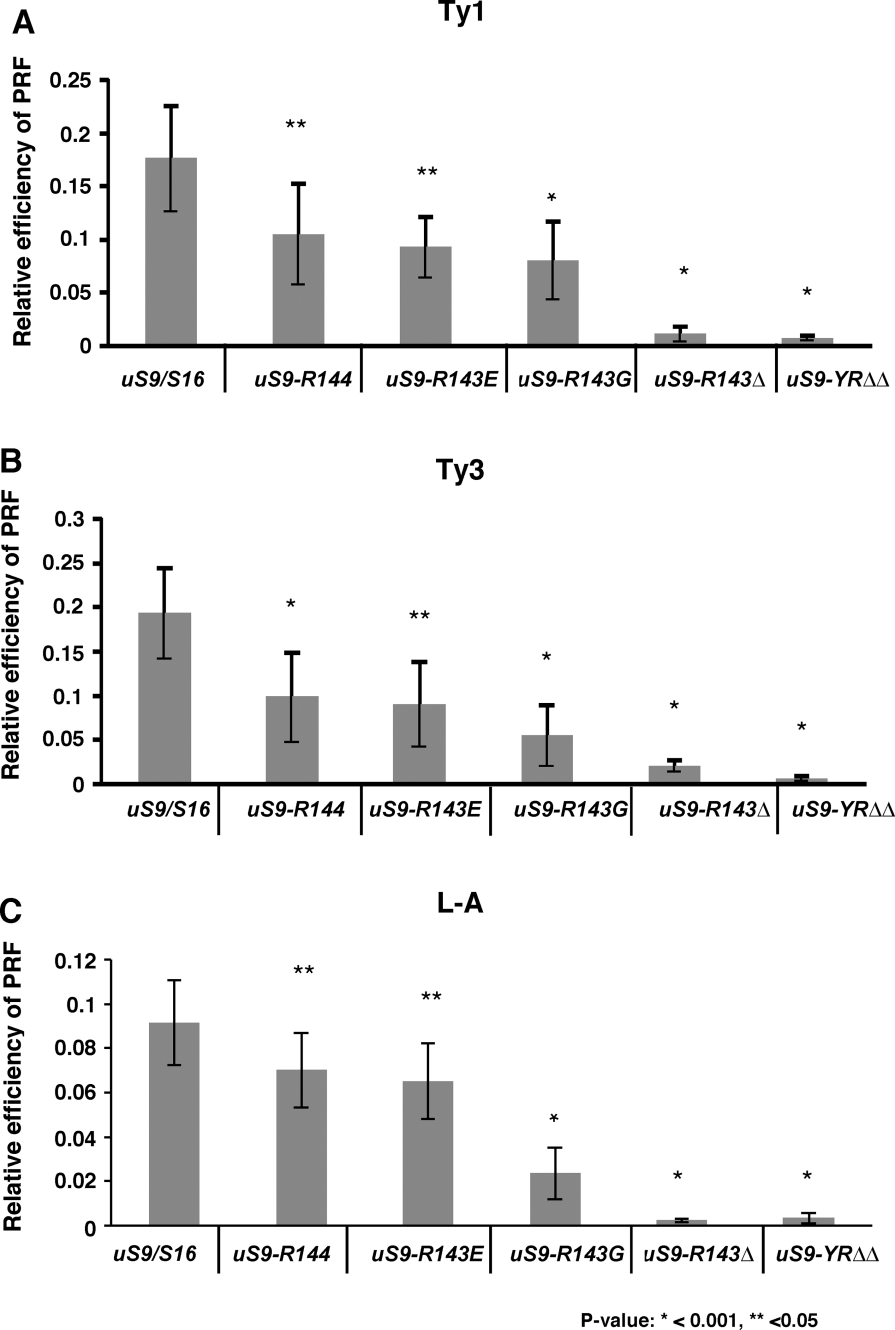
Reduced translation elongation fidelity in *uS9/S16-R143G, uS9/S16-R143Δ, uS9/S16-YRΔΔ, uS9/S16-R143E*, and *uS9/S16-R144* strains. Wild-type (WT) and mutant yeast strains were transformed with (**A**) Ty1 (+1 frameshift reporter plasmid), (**B**) Ty3 (+1 frameshift reporter plasmid), and (**C**) L-A (–1 frameshift reporter plasmid). Dual luciferase assays were performed and programmed frameshifting (PRF) efficiencies were calculated as described in Materials and Methods. Mean efficiencies (relative to WT) determined from at least three independent experiments are plotted with bars representing standard errors. The statistical significance of differences in signals between mutant and WT strains is indicated.

Plus one (+1) frameshifting happens when translating ribosomes slip one base in the 3′ direction. In the context of an elongation cycle, the slip can occur when the P-site is occupied during a ribosomal pause and A-site is empty (i.e., after translocation and before attachment of aa-tRNA in complex with eukaryotic elongation factor 1A (eEF1A) to the A-site) (38–40). Interestingly, decreased +1 programmed ribosomal frameshifting (PRF) was observed in our uS9 CTT mutant yeast strains for both Ty1 and Ty3 signals (Figure 7A and B and Supplementary Figures S5 and S6). *uS9/S16-R144, uS9/S16-R143E*, and *uS9/S16-R143G* strains showed a moderate but a clear decrease (∼2.0–4.0-fold), whereas *uS9/S16-R143Δ* and *uS9/S16-YRΔΔ* showed more substantial decreases (∼10–15-fold and ∼30-fold respectively). These results suggest that the uS9 CTT plays an important role during translocation at the P-site.

In contrast to +1 PRF, –1 PRF occurs when translating ribosomes slip by one base in the 5′ direction and involves both A- and P-sites, occupied by cognate tRNA during a ribosomal pause (38,41,42). During an elongation cycle, –1 frameshifting occurs after delivery of the cognate aa-tRNA to the ribosome and prior to the peptidyl transfer step (9). Using the dual-luciferase reporter containing an L-A virus-derived frameshifting signal, we found that –1 PRF was decreased in all uS9 mutant strains and that the relative degree of the effect in different mutant strains was similar to that observed for +1 PRF (Figure 7C and Supplementary Figures S5 and S6). Thus, *uS9/S16-R144, uS9/S16-R143E* and *uS9/S16-R143G* strains displayed moderate decreases (∼1.5–5.0-fold) while *uS9/S16-R143Δ* and *uS9/S16-YRΔΔ* strains exhibited more significant decreases of ∼45- and ∼30-fold, respectively, compared to the wild-type strain.

**Figure 8.**
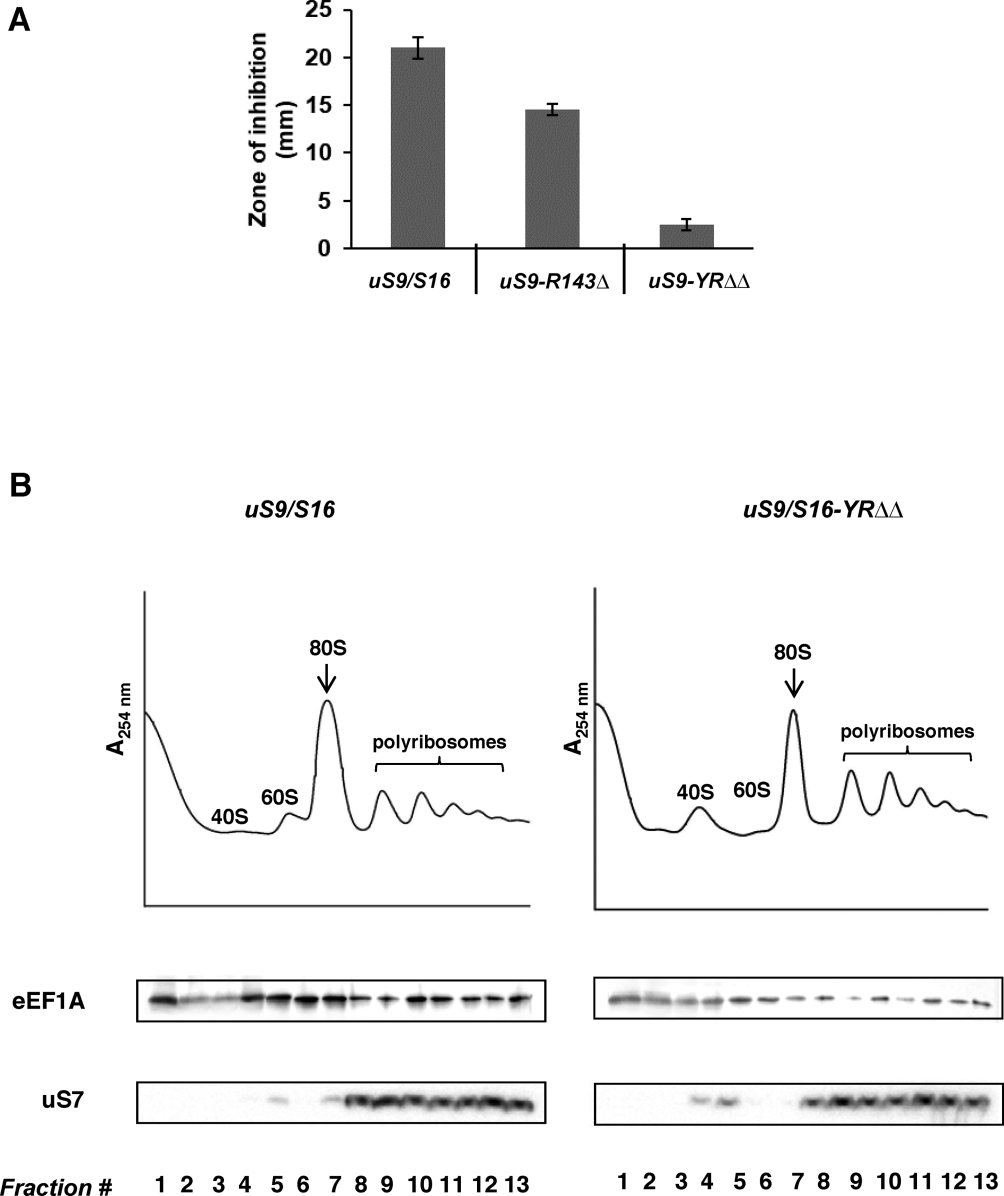
Antibiotic resistance and reduced eEF1A association of uS9 mutant yeast ribosomes. (**A**) Anisomycin resistance phenotypes of wild-type (WT) and *uS9/S16-YRΔΔ* mutant yeast strains. Overnight yeast cultures were diluted to OD _600_ = 0.3, and 300 μl of the resulting suspensions were plated onto rich medium. A 0.5 cm diameter well was created sterilely in the center of the plate and filled with 20 μg anisomycin. Plates were incubated at 30°C for 3 days and the diameters of growth inhibition zones were monitored and plotted as bar graphs. (**B**) Cell extracts were resolved by velocity sedimentation on 7–50% sucrose gradients. Fractions were collected while scanning at *A*_254_ nm, resolved by SDS-PAGE and analyzed by Western blotting using antibodies against eEF1A and uS7. The positions of different ribosomal species are indicated.

It is known that the efficiency of -1 PRF can be severely affected by alterations in the accommodation step of translation, i.e. active insertion of the 3′ end of the aa-tRNA into the ribosomal A-site by eEF1A (9). Therefore, we wanted to check if the accommodation step was affected by mutations in the uS9 CTT. Since anisomycin is an antibiotic that inhibits translation by blocking the accommodation step (9,43), we reasoned that sensitivity or resistance to anisomycin in uS9 mutants would indicate decreased or increased accommodation efficiency, respectively. Indeed, in disk agar diffusion susceptibility assays, the two strains with the most severe defects in frameshifting (*uS9/S16-R143Δ* and *uS9/S16-YRΔΔ*) showed increased resistance to anisomycin compared to the wild-type strain (Figure 8A). The reduced ability of anisomycin to block aa-tRNA insertion at the A-site may be due to higher accommodation rates in these uS9 mutants compared to the wild-type strain and/or distortion of the P-site. Given that eEF1A delivers aa-tRNA to the A-site and thereby promotes its accommodation, we looked at the polyribosomal association of eEF1A in *uS9/S16-YRΔΔ* (the uS9 mutant strain demonstrating the highest level of anisomycin resistance). Western blot analysis showed decreased eEF1A association with polyribosomes in this strain (Figure 8B). Correct codon recognition by the A-site-tRNA, triggers hydrolysis of GTP by eEF1A, followed by release of the factor and aa-tRNA accommodation at the A-site (5). Increased rates of GTP hydrolysis by eEF1A will not only cause diminished eEF1A association with polyribosomes, but should also lead to higher aa-tRNA accommodation rates which would in turn elicit increased anisomycin resistance and reduced -1 PRF (Figure 7C, Supplementary Figure S5 and Figure 8A). Therefore, our data suggest an additional role for the uS9 CTT in the elongation phase of translation: recruitment of the ternary complex (eEF1A•GTP•aa-tRNA) to the A-site and promotion of its intrinsic GTPase activity.

## DISCUSSION

Even though ribosomal proteins are not involved in the catalysis of peptide bond formation per se (44), some of them, in concert with rRNAs, build important functional regions of the ribosome (6). Several proteins (e.g. uS7, uS9, etc.) contribute to the formation of the tRNA binding sites (A, P and E) on the ribosome (6). As such, they are expected to be important regulators of key translation activities such as recruitment of translation factors and/or tRNA binding and release (30,31,45–48). Our study explored the functional significance of the C-terminal tail of uS9 at the P- and A-sites during initiation and elongation, respectively. We provide evidence showing that the uS9 CTT is important for scanning, start codon recognition and GTP hydrolysis during translation initiation and also has a potential role in regulating the correct placement of the eEF1A•GTP•aa-tRNA ternary complex at the decoding center and promoting GTP hydrolysis during translation elongation.

### The uS9 CTT promotes scanning, start codon recognition and GTP hydrolysis in 43/48S PICs during translation initiation

From available structural data, particularly of yeast ribosomes, it is clear that uS9/S16 is located on the solvent side of the small (40S) ribosomal subunit head and has a long protruding C-terminal tail (CTT) that contacts the initiator tRNA base-paired to the AUG codon in the P-site (49) (Figure 1A). High resolution structures of eukaryotic ribosomal complexes have further revealed that the last C-terminal arginine (Arg-143) residue of uS9 (which is invariably conserved in all kingdoms of life) interacts with the negatively charged initiator tRNA at the P-site (30,31). While it was found using bacterial cell systems that the uS9 CTT may be an important regulator of translation initiation fidelity (50), the mechanism of its action was not evident and similar studies in eukaryotic cells were lacking. We previously determined that the terminal arginine (Arg-143) and the penultimate tyrosine (Tyr-142) of uS9 are important for efficient translation initiation and reinitiation in the yeast *S. cerevisiae* (7). However, a molecular understanding of this effect was still lacking. To further investigate the role of uS9 C-terminal residues in translation initiation, we generated additional *S. cerevisiae* mutant strains expressing mutant uS9 variants containing CTT extensions and/or substitution of the C-terminal Arg with a negatively charged residue (Glu). Glutamate substitution of Arg-143 (*uS9/S16-R143E*), addition of an extra arginine after Arg-143 (*uS9/S16-R144*) and deletion of both Arg-143 and Tyr-142 (*uS9/S16-YRΔΔ*, shown previously (7)) confer a Slg− phenotype (Figure 2A). This suggests that not only the length of uS9 CTT, but also the nature of the (positive) charge at the terminal residue may play an important role in yeast cell physiology, and likely in key steps of translation. Further, *uS9/S16-R143E, uS9/S16-R144* and *uS9/S16-YRΔΔ* strains showed a reduced rate of bulk translation initiation, as evidences by increased levels of 80S ribosomes and concomitant reduction of polyribosomes (Figure 2B) (7). The results of polyribosomal analysis were further corroborated by analysis of translation reinitiation defects. We demonstrated that impaired *GCN4* mRNA translation in response to amino acid limitation was exhibited not only by *uS9/S16-R143E, uS9/S16-R144* and *uS9/S16-YRΔΔ* strains, but also by *uS9/S16-R143Δ* and *uS9/S16-R143G* with Arg-143 deletion or substitution with Gly (Figure 3A and Supplementary Figure S2), even though the latter two mutants did not show any growth defects or significantly reduced bulk translation initiation (Figure 2) (7). Since *GCN4* mRNA translation depends on efficient reinitiation at the AUG of *GCN4* ORF (after bypassing the inhibitory uORFs 2–4 in the *GCN4* mRNA leader), the defect observed in this process in all of the uS9 mutants clearly demonstrates the importance of the positively charged terminal arginine and penultimate tyrosine of the uS9 CTT during translation initiation.

To understand the mechanism(s) underlying the phenotypes of the tested uS9 mutants, we performed in-depth analysis of different translation initiation steps using both *in vivo* and *in vitro* approaches. Addition of an extra Arg at the C-terminus of uS9 gave rise to the most severe phenotypic defects of Slg−, Gcn− and a reduced rate of bulk translation initiation (Figures 2 and 3A and Supplementary Figure S2). This mutation (*uS9/S16-R144*) was further analyzed using a panel of *GCN4*-lacZ reporters harboring different arrangements of uORFs. The results of these experiments led us to conclude that the Gcn− phenotype of the *uS9/S16-R144* mutant is likely due to: (i) inability of its 40S ribosomal subunits to resume scanning after terminating translation at uORF1 (Figure 3B and Supplementary Figure S3), and/or (ii) failure to recognize the AUG codon at uORF1 (leaky scanning) (Figure 4A and Supplementary Figure S3). Whereas in *uS9/S16-R143E* strain, defective resumption of scanning by 40S ribosomal subunits (after translating uORF1) confers the Gcn− phenotype (Figure 3, Supplementary Figures S3 and S4). Compromised *GCN4* mRNA translation in *uS9/S16-R143G* and *uS9/S16-YRΔΔ* mutants can be attributed to their strong leaky scanning phenotype (Figure 4A and Supplementary Figure S3) (7). Defective resumption of scanning in *uS9/S16-R144* and *uS9/S16-R143E* is likely due to inability of the mutant 40S ribosomal subunits to remain attached to the *GCN4* mRNA during reinitiation (51). These data suggest that the exact position of the last uS9 CTT residue and the nature of its charge are critical during the scanning phase of general translation initiation. More specifically, our results indicate that the positively charged C-terminal Arg of uS9 modulates binding of the TC to 40S subunits in 43S scanning preinitiation complexes.

Having observed the strong leaky scanning phenotype in uS9 mutants, we sought to confirm the role of the uS9 CTT in start codon recognition. In *HIS4-lacZ* reporter assays, utilization of both cognate and non-cognate codons was reduced in *uS9/S16-YRΔΔ, uS9/S16-R143E* and *uS9/S16-R144* strains exhibiting the most severe defects (Figure 4B and C and Supplementary Figure S3). This is consistent with our previous observation that most of the uS9 mutants showed inefficient recognition of the uORF1 start codon during translation of the *GCN4* reporter construct pM226 (Figure 4A and Supplementary Figure S3). Interestingly, uS9 mutations caused greater reduction in UUG- versus AUG codon initiation which is compatible with the fact that UUG codon mismatches with the anticodon of tRNA_i_, already disfavor this event. Further evidence supporting a role for the uS9 CTT in 48S PIC formation was the demonstration of increased association of eIF1 and eIF2 with 40S ribosomal subunits (Figure 5). Increased accumulation of eIF1 and eIF2 was observed in the majority of the mutants with *uS9/S16-R143E* and *uS9/S16-R144* showing the highest levels of accumulation (Figure 5). eIF1 and eIF2 are known to be displaced from the initiation complex after establishment of correct codon–anticodon base pairing (4). Hence, it is conceivable that increased amounts of eIF1 and eIF2 bound to 40S ribosomal subunits could arise from an upstream defect in scanning by the preinitiation complex such as AUG recognition (discussed above) or GTP hydrolysis (52,53). Overall, our data show that the optimal CTT length and positive charge of Arg-143 are required for proper formation and function of 43/48S PICs.

According to the current model of scanning initiation, binding of the 43S complex to mRNA accelerates structural rearrangement allowing GTPase activating protein (GAP) to stimulate GTP hydrolysis on eIF2 and establishment of an internal equilibrium between GTP and GDP•Pi. eIF1 does not inhibit GTP hydrolysis itself but regulates release of Pi from eIF2•GDP•Pi. As soon as the correct start codon–anticodon base pairing is established, conformational changes accompany eIF1 release, which further regulates the release of Pi from eIF2•GDP•Pi (3,24). Once GTP is hydrolyzed irreversibly, the affinity of eIF2 for Met-tRNA_i_^Met^ is reduced, leading to partial dissociation of eIF2•GDP from 40S subunits (52,53). Thus, accumulation of eIF1 and eIF2 bound to 40S subunits in the tested uS9 mutants (as described above) implies restricted GTP hydrolysis and/or Pi release with a delay in the conformational rearrangement from the open/P_OUT_ configuration to the closed/P_IN_ state. Preliminary evidence suggesting a role of the uS9 CTT in GTP hydrolysis is that the Slg− phenotype (7) and accumulation of eIF2 on 40S ribosomal subunits observed in *uS9/S16-YRΔΔ, uS9/S16-R143E* and *uS9/S16-R144* mutants were both mitigated by introducing the dominant *SUI5* allele encoding eIF5-G31R into these strains (Figure 6A). This (eIF5-G31R) variant acts as a GAP and also regulates gated Pi release from eIF2•GDP•Pi (54). If uS9 CTT mutations impair GTP hydrolysis and/or Pi release in the scanning complex, then it could be proposed that introducing eIF5-G31R into the above mutants restores a near wild type rate of GTP hydrolysis that accounts for the suppression of uS9 mutant phenotypes by SUI5. GTPase assays using a fully reconstituted yeast initiation system containing eIFs (1, 1A, 2 and 5) and a model mRNA provided support for this hypothesis. Deletion of the last two CTT residues or substitution/addition of the terminal arginine by glutamate and arginine respectively, led to a reduced rate of GTP hydrolysis (Figure 6B). It should be noted that this assay is extremely sensitive. It was previously shown that an eIF5-R15M GAP mutant didn’t activate GTP hydrolysis even when used much above physiological concentrations (24). Thus, we propose that the uS9 CTT is involved in events surrounding eIF5-stimulated GTP hydrolysis within the eIF2•GTP•Met-tRNA_i_^Met^ complex. However, since this assay cannot distinguish between ‘irreversible hydrolysis’ in which Pi has been released and ‘internal hydrolysis’ in which an equilibrium between GTP and GDP•Pi has been established but Pi has not yet been released, whether uS9 CTT mutations affect gated Pi release in the PIC remains unclear. Although it is conceivable that during structural rearrangement (accelerated due to binding of the 43S complex to mRNA (24)) the uS9 CTT might be involved in stabilizing the transition state for GTP hydrolysis, a role in gated Pi release from eIF2•GDP•Pi cannot be ruled out. Support for the latter possibility includes the observation that expressing eIF5-G31R in uS9 CTT mutants reversed the accumulation of eIF2 on native 40S subunits, which might not appear unless Pi was released to allow eIF2•GDP to dissociate from the 40S complex. We note, however, that while the overall consequences (impaired initiation and reinitiation, compromised GTP hydrolysis) of the uS9 mutations under investigation could be well documented, the exact contribution of each particular mutation to the observed phenotypes will require detailed structural analysis of the molecular environment of the uS9 CTT in the mutant strains.

### Functions of the uS9 CTT during translation elongation

The uS9 CTT mutations evaluated in this study all reduced +1 (Ty1 and Ty3) and -1 (L-A) programmed ribosomal frameshifting (PRF), with the most severe effects observed in *uS9/S16-YRΔΔ* and *uS9/S16-R143Δ* strains (Figure 7 and Supplementary Figures S5 and S6). Interestingly, the increased fidelity observed in these experiments was contrary to the reduced fidelity exhibited by the same mutants during studies of translation initiation (Figure 4 and Supplementary Figure S3). Since +1 frameshifting takes place after translocation at the P-site and before accommodation at the A-site (38), reduced +1 PRF in uS9 mutants could occur because of incomplete translocation, suggesting a possible role for the uS9 CTT in modulating P-site tRNA positioning during the elongation cycle. On the other hand, –1 PRF occurs only after delivery of aa-tRNA at the A-site or completion of the accommodation step (38). Anisomycin resistance and reduced association of eEF1A to polyribosomes (Figure 8) correlated with severely compromised –1 PRF efficiency in the *uS9/S16-YRΔΔ* mutant strain. One possible explanation for this observation is that GTP hydrolysis during the elongation phase is altered in this mutant. Increased rates of intrinsic or facilitated GTP hydrolysis in this mutant could account for increased aa-tRNA accommodation rates and thus resistance to anisomycin. Increasing the intrinsic ability of eEF1A to accommodate the aa-tRNA into the A-site of the peptidyl transferase center by the mutant uS9 would overcome the blocks imposed by anisomycin at this step and would eventually lead to dissociation of eEF1A from the ribosomes (as seen in Figure 8B). In addition, increasing aa-tRNA accommodation rates would decrease the amount of time that ribosomes would be paused at the –1 frameshift signal, decreasing the likelihood of slippage and the rate of –1 PRF. Similar effects have been observed in an eEF1A mutant (N153T) with increased GTPase activity, reduced -1 PRF and increased resistance to anisomycin like antibiotic (8,9). It is possible that mutations in the uS9 CTT might affect physical transduction of signals from the A-site through the aa-tRNA to the eEF1A GTPase center. Changes in the uS9 CTT may alter the architecture of the P-site, causing its distortion. This, in turn, could alter kinetic partitioning during impeded translocation and cause –1 PRF (55,56). While the mechanism of –1 PRF is not entirely clear, it is generally accepted that kinetic partitioning at a slippery site explains for why and when it occurs (55,56). Recent data suggest that –1 PRF can occur simultaneously through several pathways (55). It has been suggested, however, that kinetic parameters of aa-tRNA binding, peptide bond formation, and translocation are the key to this event (55,56). Thus, altered kinetic partitioning is a likely cause for the decreased –1 PRF observed in mutant uS9 strains. Nevertheless, additional biochemical and structural analyses are required for an in depth molecular understanding of the process in the mutant yeast strains.

### Concluding remarks

**Figure 9.**
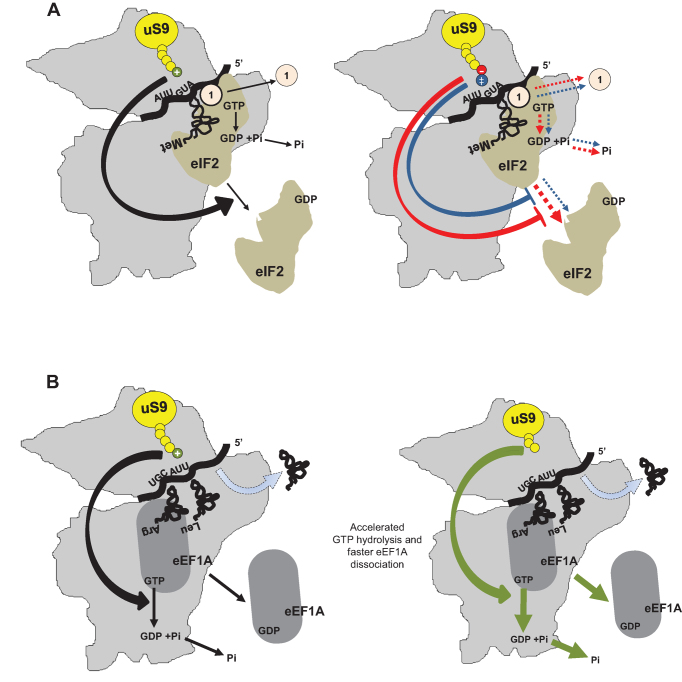
Proposed model for uS9 C-terminal tail involvement in initiation and elongation processes in eukaryotes. (**A**) Initiation: Left—under wild-type conditions, proper positioning of the AUG start codon in the P-site is influenced by the correct location of the uS9 CTT and the charge of the last C-terminal positively charged Arg. The CTT triggers efficient eIF2-bound GTP → GDP + Pi hydrolysis, followed by optimal dissociation of eIF1 and eIF2 from the 48S complex. Right—reversal of the CTT C-terminal charge (red, –, *uS9/S16-R143E* mutant) and/or addition of an extra Arg (positive charge) (blue, ‡, *uS9/S16-R144* mutant) results in inefficient GTP hydrolysis and compromised release of eIF1 and eIF2 from the complex. The severity of the effects of each mutation are reflected in the thickness of the dashed lines (thicker lines represent more severe defects while thinner lines represent less severe defects). (**B**) Elongation: Left - under wild-type conditions, the uS9 CTT mediates cooperation between the ribosomal P- and A-sites, promoting efficient eEF1A-mediated GTP hydrolysis and tRNA accommodation, followed by optimal dissociation of eEF1A. Right—deletions (*uS9/S16-YRΔΔ* mutant) and/or mutations in the CTT reduce cooperation between the P- and A-sites and result in more stringent tRNA selection/accommodation during elongation accompanied by enhanced eEF1A bound GTP-hydrolysis and dissociation of eEF1A from elongating 80S ribosomes.

Our combined genetic and biochemical analysis of uS9 CTT mutants demonstrated that during translation initiation, the appropriate length and charge of the uS9 CTT are critical for a number of events downstream of 43S and 48S complex assembly, particularly recruitment of the TC, scanning, AUG recognition, and GTP hydrolysis at the P-site (Figure 9A). Whether this region of uS9 is directly involved in each of the above processes or affects an initial upstream event influencing the remaining downstream steps is unclear at this stage. Furthermore, we found that the uS9 CTT is also important during the elongation phase of translation, possibly regulating translocation at the P-site and tRNA stabilization/accommodation with GTP hydrolysis at the A-site (Figure 9B). It is important to mention that while uS9 CTT mutations clearly reduce the fidelity of initiation, their effects on elongation are opposite as they increase the stringency of decoding. Therefore, the uS9 CTT may operate through distinct mechanisms during initiation and elongation phases, potentially due to formation of different sets of contacts with initiator tRNA versus elongator tRNAs (Figure 9). In this context, increased GTPase activity in uS9 CTT mutant strains during elongation makes sense, which contrasts with the reduced GTP hydrolysis seen during initiation. Overall, the results of this study suggest that the uS9-CTT has evolved specifically to increase the fidelity and efficiency of initiation rather than fidelity during elongation.

## Supplementary Material

Supplementary Data
