# Supplementary material for: Role of the uS9/yS16 C-terminal tail in translation initiation and elongation in Saccharomyces cerevisiae
Source: Nucleic Acids Res. 2018 Nov 27;47(2):806–23. doi: 10.1093/nar/gky1180 (PMC6344880; doi:10.1093/nar/gky1180)
Supplement: Supplementary Data [file gky1180_supplemental_files.pdf]

SUPPLEMENTAL MATERIAL

FIGURE LEGENDS

Figure S1. rRNA analysis from wt and mutant yeast strains. Total yeast RNA was separated on a denaturing agarose gel and stained with ethidium bromide. The positions of 25S and 18S rRNA species and the 18S/25S rRNA ratios are indicated.

Figure S2. Gcn phenotypes of WT and uS9/rps16 mutant yeast strains. Yeast cell growth. Serial dilutions of strains spotted onto minimal media under non-starved (-3-AT), or amino acid (aa) starved conditions (+3-AT), respectively.

Figure S3. Expression of reporter *GCN4-lacZ* constructs in WT and mutant yeast strains. β-Galactosidase activity (units)/raw values as presented in Fig. 3A measured under normal (-SM) and amino acids starved conditions (+SM) are shown. The standard errors are shown in parentheses.

Figure S4. Expression of reporter *lacZ* constructs in WT and mutant yeast strains. β-Galactosidase activity (units)/raw values as presented in Fig. 3B and Fig. 4 measured under normal are shown. The standard errors are shown in parentheses.

Figure S5. Relative values of reporter frameshift L-A (pJD376), Ty1 (pJD377) and Ty3 (pJD379) constructs expression in WT and mutant yeast strains. Values as presented in Fig. 7. The standard errors are shown in parentheses.

Figure S6. Expression of reporter frameshift L-A (pJD376), Ty1 (pJD377), Ty3 (pJD379) constructs and a control pJD375 contsruct (not containing any frameshift signals) in WT and mutant yeast strains. Firefly and Renilla luciferase activities (units)/raw values are shown (for the values presented in Fig. S5). The standard errors are shown in parentheses.

Figure S1

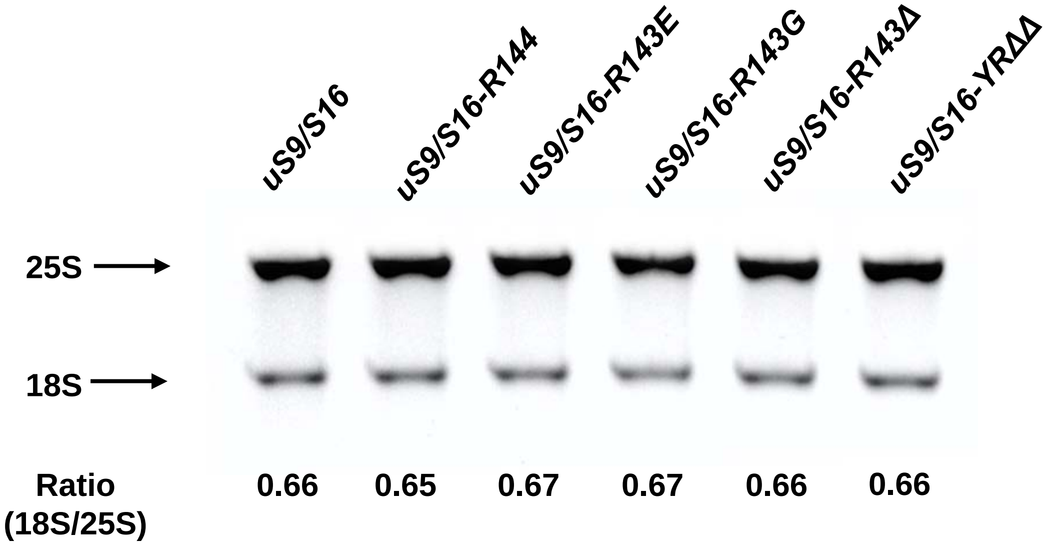
uS9/S16
uS9/S16-R144
uS9/S16-R143E
uS9/S16-R143G
uS9/S16-R143Δ
uS9/S16-YRΔΔ
25S
18S
Ratio (18S/25S)
0.66
0.65
0.67
0.67
0.66
0.66

Figure S2

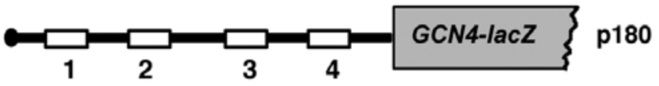

Strain \ Construct	p180	
	**-3AT**	**+3AT**
uS9/S16	**53.70** (±2.31)	**311.31** (±28.36)
uS9-R143G	**44.23** (±3.54)	**114.3 *** (±3.91)
uS9-R143Δ	**45.7** (±18.96)	**72.11 *** (±16.41)
uS9-YRΔΔ	**34.44 *** (±13.82)	**41.03 *** (±6.00)
uS9-R143E	**8.04 *** (±2.32)	**6.45 *** (±6.00)
uS9-R144	**18.84 *** (±1.48)	**70.81 *** (±22.35)

P -value: * = <0.001

Figure S3

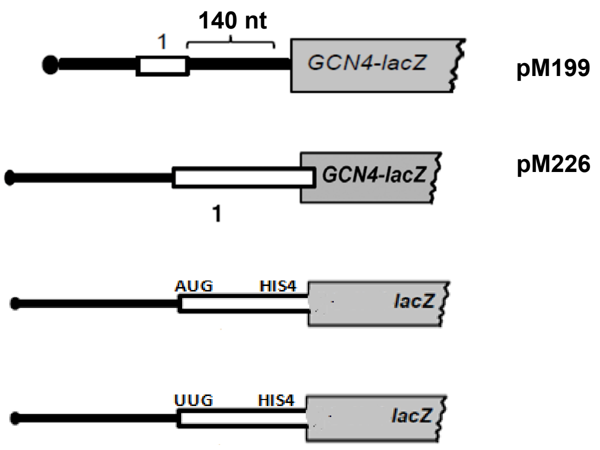

Reporter plasmid	β-gal activity (U)						
	3AT	*uS9/S16*	*uS9-R143G*	*uS9-R143∆*	*uS9-YR∆∆*	*uS9-R143E*	*uS9-R144*
**pM199**	-	**1041.25** (±125.86)	**1004.60** (±125.97)	**1110.80** (±203.14)	**952.89** (±78.42)	**600.02 *** (±133.63)	**444.70 *** (±109.13)
**pM226**	-	**1.636** (±0.41)	**144.49 *** (±11.06)	**0.053 *** (±0.07)	**4.00 *** (±0.97)	**2.17** (±0.37)	**30.08 *** (±3.22)
**AUG**	-	**390.25** (±20.77)	**195.91 *** (±33.69)	**175.60 *** (±30.88)	**102.26 *** (±5.7)	**101.50 *** (±17.66)	**107.76 *** (±28.5)
**UUG**	-	**5.88** (±0.98)	**2.10 *** (±0.16)	**1.89 *** (±0.45)	**1.47 *** (±0.01)	**0.75 *** (±0.12)	**0.72 *** (±0.13)

P -value: * = <0.001

Figure S4

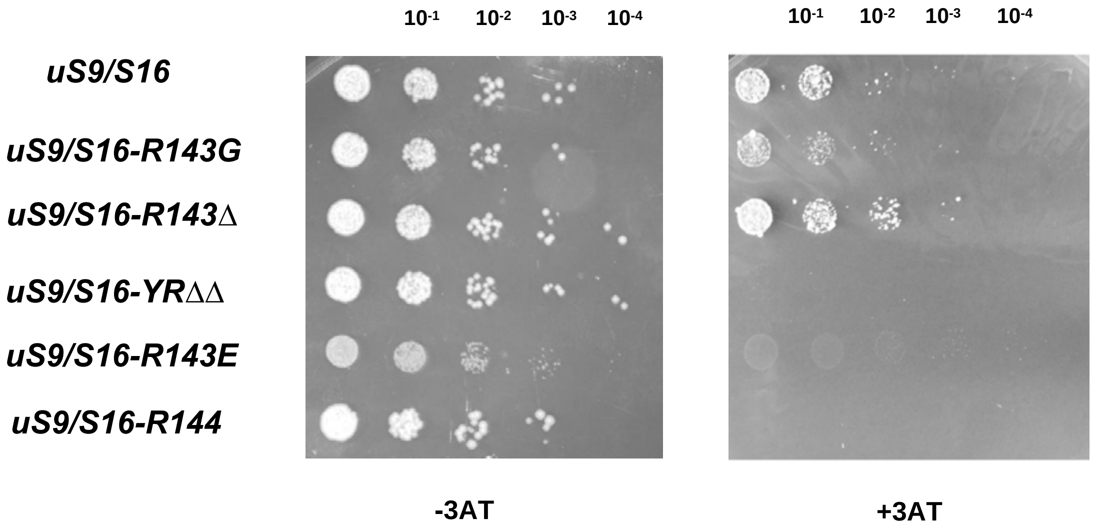
10-1
10-2
10-3
10-4
10-1
10-2
10-3
10-4
uS9/S16
uS9/S16-R143G
uS9/S16-R143Δ
uS9/S16-YRΔΔ
uS9/S16-R143E
uS9/S16-R144
-3AT
+3AT

Figure S5

Reporter / Strain	L-A (pJD376) (-1 frameshift)	Ty1 (pJD377) (+1 frameshift)	Ty3 (pJD379) (+1 frameshift)
uS9/S16	**0.091** (±0.019)	**0.176** (±0.050)	**0.193** (±0.052)
uS9-R144	**0.007 **** (±0.017)	**0.105 **** (±0.048)	**0.098 *** (±0.051)
uS9-R143E	**0.065 **** (±0.017)	**0.092 **** (±0.029)	**0.090 **** (±0.049)
uS9-R143G	**0.024 *** (±0.012)	**0.080 *** (±0.037)	**0.055 *** (±0.035)
uS9-R143Δ	**0.003 *** (±0.001)	**0.011 *** (±0.008)	**0.020 *** (±0.007)
uS9-YRΔΔ	**0.004 *** (±0.019)	**0.006 *** (±0.003)	**0.006 *** (±0.003)

P-values: * < 0.001, ** <0.05

Figure S6

Reporter	Yeast Strain	Firefly luciferase	Renilla luciferase	Ratio (F-luc/R-luc)
**pJD377** (+1 frameshift) **(Ty1)**	***uS9/S16***	**296013** (±32435)	**22645640** (±1550970)	**0.0132** (±0.0011)
	uS9-R144	**98646** (±13932)	**10828468** (±1014873)	**0.0096** (±0.0012)
	uS9-R143E	**188341** (±28744)	**19794674** (±19794674)	**0.0089** (±0.0008)
	uS9-R143G	**110698** (±25821)	**8303234** (±1879711)	**0.0127** (±0.0018)
	uS9-R143Δ	**171520** (±50810)	**12232004** (±2794529)	**0.0101** (±0.0022)
	uS9-YRΔΔ	**95642** (±28643)	**11933393** (±3130046)	**0.0062** (±0.0009)
**pJD379** (+1 frameshift) **(Ty3)**	***uS9/S16***	**360282** (±51524)	**25549623** (±3005689)	**0.0145** (±0.0012)
	uS9-R144	**35973** (±3294)	**4883881** (±704420)	**0.0092** (±0.0014)
	uS9-R143E	**43771** (±5372)	**5897086** (±722708)	**0.0087** (±0.0014)
	uS9-R143G	**150771** (±36460)	**12702546** (±2696322)	**0.0087** (±0.0017)
	uS9-R143Δ	**503683** (±83463)	**25348867** (±1770350)	**0.0189** (±0.0022)
	uS9-YRΔΔ	**104259** (±26181)	**15872672** (±3119853)	**0.0057** (±0.0008)
**pJD376** (-1 frameshift) **(L-A)**	***uS9/S16***	**61070** (±3175)	**9090018** (±612490)	**0.0068** (±0.0004)
	uS9-R144	**20472** (±1923)	**3268999** (±339044)	**0.0064** (±0.0004)
	uS9-R143E	**39021** (±3294)	**6356722** (±521134)	**0.0063** (±0.0005)
	uS9-R143G	**31712** (±4541)	**10696160** (±1864324)	**0.0037** (±0.0005)
	uS9-R143Δ	**29349** (±5650)	**12962683** (±2592137)	**0.0023** (±0.0001)
	uS9-YRΔΔ	**17508** (±6099)	**5594329** (±1035086)	**0.0034** (±0.0007)
**pJD375** (control)	***uS9/S16***	**1086015** (±130025)	**14177618** (±1215248)	**0.0751** (±0.0046)
	uS9-R144	**449928** (±75599)	**4834528** (±535435)	**0.0971** (±0.0128)
	uS9-R143E	**976693** (±183012)	**10176969** (±1297007)	**0.0921** (±0.0173)
	uS9-R143G	**3005301** (±373924)	**19325806** (±2500424)	**0.1603** (±0.0119)
	uS9-R143Δ	**4083** (±662)	**4075** (±510)	**0.9554** (±0.0526)
	uS9-YRΔΔ	**1641** (±220)	**1750** (±255)	**0.9742** (±0.0531)
